# Changes in gene expression and metabolic profile of drupes of *Olea europaea* L*.* cv Carolea in relation to maturation stage and cultivation area

**DOI:** 10.1186/s12870-019-1969-6

**Published:** 2019-10-16

**Authors:** Leonardo Bruno, Ernesto Picardi, Marianna Pacenza, Adriana Chiappetta, Antonella Muto, Olimpia Gagliardi, Innocenzo Muzzalupo, Graziano Pesole, Maria Beatrice Bitonti

**Affiliations:** 1 Dipartimento di Biologia, Ecologia e Scienze della Terra, Università della Calabria, 87036 Arcavacata di Rende (CS), Italy; 2 Department of Biosciences, Biotechnology and Biopharmaceutics, University of Bari A. Moro, Bari, Italy; 3 Institute of Biomembranes, Bioenergetics and Molecular Biotechnologies, Consiglio Nazionale delle Ricerche, Bari, Italy; 4 Centro di Ricerca per l’Olivicoltura-Frutticoltura-Agrumicoltura (OFA) Consiglio per la Ricerca in agricoltura e l’analisi dell’Economia Agraria (CREA) C.da Li Rocchi-Vermicelli, 87036 Rende (CS), Italy

**Keywords:** *Olea europaea* L*.*, Drupe maturation, Cultivation area, Transcriptome, Metabolic profile

## Abstract

**Background:**

Olive (*Olea europaea* L.) is an emblematic oil tree crop in the Mediterranean basin. Currently, despite olive features as a moderately thermophilic species, its cultivation is worldwide spreading due to the health-related impact of olive products on human nutrition. A point of concern for the expanding olive cultivation is related to the influence that, in addition to genotype, environmental factors exerts on drupe development and metabolism with consequent impact on fruit key traits. In this context, the aim of the present work was to gain further information on the genetic networks controlling drupe maturation phase and, mainly, on their modulation in response to environmental cues.

**Results:**

To achieve this goal, a comparative transcriptome-wide investigation was carried out on drupes of *Olea europaea* cultivar Carolea, collected from plants growing in areas at different altitude level and therefore experiencing different climatic conditions. Two maturation stages of drupe were analysed: green mature and turning-purple. Metabolic characterization of drupe was also performed. At both transcriptomic and metabolic level differences were detected in the pathway of fatty acids (FAs) and phenol compounds, in relation to both drupe maturation stage and cultivation area. Among the most relevant differences detected during the transition from GM to TP stages there were: the upregulation of FADs genes in the drupes of population growing at 700 masl, the upregulation of phenol biosynthesis-related genes in drupes growing at 10 and 200 masl and very interestingly the downregulation of specific genes involved in secoiridoids production in drupes growing at 700 masl. Globally, these results suggested that stability of FAs and phenols, mainly of secoiridoids group, is promoted at high altitude, while at lower altitude phenol biosynthesis is prolonged.

**Conclusion:**

The obtained results showed a differential modulation of genetic pathways related to olive compound quality in relation to the cultivation area, likely imposed by the different temperature impending at each altitude. The derived molecular information appears of interest for both breeding and biotechnological programs of olive species, especially with respect to the modulation of antioxidant secoiridoid compounds which play a key role in conferring both sensorial and healthy characteristic to olive products.

**Electronic supplementary material:**

The online version of this article (10.1186/s12870-019-1969-6) contains supplementary material, which is available to authorized users.

## Background

Olive (*Olea europaea* L.) is one of the oldest tree crop species growing in the Mediterranean basin and represents the sixth most important oil fruit crop in the world [1, 2]. In addition, the healthy effects of olive products have been largely assessed, thus leading to a strong impact of olive cultivation on the human nutrition [3, 4]. Accordingly, in the last decades olive oil consumption has worldwide expanded [5] and olive tree cultivation extended also in non-traditional producer countries, outside the Mediterranean area [6, 7].

In this context of important concern is the deep influence that environmental factors exert, in addition to genotype, on the physiological processes and metabolic pathways underlying the olive fruit (drupe) development, which is characterized by accumulation in the pericarp of oil, and several minor components exhibiting antioxidant activity [8–11]. As a result, the environment can strongly impact on the quality and the healthy properties of olive products (i.e. fruit and oil), which rely on both acyl composition, characterized by a low content of saturated fatty acids (FAs), and the presence of such antioxidant metabolites.

In particular, although the pattern of FAs synthesis and desaturation varies enormously among cultivars, olive oil is typically enriched in the monounsaturated oleic acid (C18:1) followed by linoleic acid (C18:2), whose percentages can reach up to 75–80% and 3.5–21% of total FAs, respectively; while palmitic acid (C16:0), stearic acid (C18:0) and linolenic acid (C18:3) represent minor components [8]. Notably, most of olive oil (> 90%) is produced in the fruit rather than in the seed [12] and accumulate in large oil bodies developed in the mesocarp cells, following a well defined time-course [13, 14].

As for the antioxidant metabolites accumulated in the drupe throughout its development, they include phenols [9, 10], carotenoids [15], and tocopherols [11]. All these compounds play a role in protecting biological macromolecules such as DNA, proteins and lipids from oxidative damage due to their radical scavenger activity. Therefore, beside conferring resistance to oxidation and oxidative stability to olive products, these minor components have relevant healthy value [2, 16–18]. At this respect, phenols which are responsible for the agreeable sensory properties of olive oil [10] have been the most studied compounds [9, 19, 20]. In particular, among them a major role is credited to the secoiridoids, which are exclusively present in the *Oleaceae* family and represent the most important class of the olive phenolics [9, 20].

Based on olive agronomic relevance, physiological processes and metabolic pathways underlying drupe development, from growth to maturation and ripening, have been largely investigated and clarified [21, 22]. However, information on the genetic networks controlling these processes and, above all, on their environment-dependent modulation is further needed in order to implement biotechnological programs addressed to improve quality and healthy characteristics of olive products. Namely, it has been largely assessed that both temperature and light play a role in modulating the balance between saturated and unsaturated FAs [14, 23] as well as the levels of antioxidants and active bio-molecules resulting from the catabolic and anabolic processes taking place through-out drupe development [24–26].

Currently, transcriptome-wide investigations carried out in different cultivars of olive provided some relevant information on the structure and putative function of genes expressed in the fruit and of potential relevance in regulating its metabolism, development and maturation [21, 27]. In addition, the recent sequencing, assembly and annotation of olive genome provides a valuable resource for gain further insight into the genetic basis underlying physiological and developmental process and key phenotypic traits of olive plants [28, 29].

In this context, focusing on the environment influence, we applied a high-throughput sequencing technology to perform a comparative transcriptomic analysis on drupes of *Olea europaea* cultivar Carolea growing at different altitude levels (i.e. 10, 200, 700 m above sea level, masl) and therefore experiencing different climatic conditions. Cultivar Carolea was selected because of its widespread cultivation in the Calabria region (Italy) for both oil and olive production. Two different maturation stages were analysed: the green mature (GM) and the turning purple (TP) stages corresponding to the stages usually used for oil and olive production. Such approach was expected to give insight into the influence of climatic conditions and mainly temperature on the gene expression pattern and fruit metabolic response, which could be relevant for the expanding cultivation area of olive plants outside traditional countries.

## Methods

### Plant material

Ten/eleven year-old propagated plants of *Olea europaea* cultivars Carolea, were used. In details, we used:

- a clonal population belonging to the olive germplasm collection of the (CREA-OFA) at Mirto Crosia (Cosenza, Calabria, Italy, latitude 39°37′04.57″N; longitude 16°45′42.00″E, altitude 10 masl);
- a clonal population belonging to the olive germplasm collection of the CREA-OFA at Rende (Cosenza, Calabria, Italy) latitude 39°21′58″N; longitude 16°13′44″E, altitude 200 masl);
- a clonal population belonging to a CREA-OFA experimental field at Morano Calabro (Cosenza, Calabria, Italy, latitudine 39°50′55.0“N; longitude 16°09’06.5”E, altitude 700 masl).

Preliminarly these three clonal populations have been characterized through microsatellite analysis as belonging to Carolea cultivar (data not shown).

From now on, these three clonal populations will be referred to as populations at 10, 200 and 700 masl, respectively.

The three populations were subjected to the same agronomic practices without irrigation, and sampling was performed in the season 2012/2013 at 150 and 180 Days After Flowering (DAF). Climatic parameters such as temperature and rain were daily registered in the different cultivation sites, indicated as 10 masl, 200 masl and 700 masl, by a WD 2700 weather station. The mean month values ± standard error are shown in Additional file 1: Figure S1.

For each population and at each sampling, at least 4000 drupes (about 20Kg) were sampled from ten different individuals (*n* = 400 for each individual). In order to minimize the effects related to the asynchronous maturation of fruits within the same tree, drupes were hand-picked from all around the external parts of the canopy of trees.

For each sampling, one part of collected drupes (10-15Kg) was immediately used for olive oil virgin extraction; one part (*n* = 300) was used for ripeness index evaluation; the remaining part (*n* = 700) was immediately fixed in liquid nitrogen, stored at − 80 °C and used for both biochemical and molecular analyses. In order to minimise individual variation and reduce variability, sample pooling methodology was applied in all the analyses as an alternative approach to biological replicates [30, 31].

### Index of ripeness of the drupes

Ripeness was determined according to the guidelines of the Spanish National Institute of Agronomic Research based on a subjective evaluation of the olive skin and pulp colours [32]. The procedure consists of distributing a randomly taken sample of 100 fruits into eight groups: 0 = drupes with epicarp green; 1 = drupes with epicarp light green; 2 = drupes with epicarp green-yellow and red traces in the distal part of the fruit, covering a quarter of the surface (beginning veraison); 3 = drupes with epicarp reddish or burnished for more than half of the surface (the end of veraison); 4 = drupes with black epicarp and clear pulp; 5 = drupes with brownish black epicarp and the pulp to less than half the depth; 6 = drupes with black epicarp and pulp browning for more than half of the depth but getting to the endocarp; 7 = drupes with black epicarp and pulp browning up to the endocarp. The maturity or Ripening Index (RI) was calculated using the following equation:

$$ \mathrm{RI}=\sum i\ast ni/100 $$

where *i* is the group number and *ni* is the number of drupes in the group. This method involves manually separating the drupes, cutting the pulp to examine it, counting them and identifying the group to which they belong. The RI values range from 0 to 7.

### Fat content in olive drupe

For each sampling, a pool of − 80 °C-stored drupes (*n* = 100) was homogenised and dried. 2-5 g aliquot of dried powder was submitted to Soxhlet extraction with 250 ml of petroleum ether for 6 h. After solvent evaporation, the flask containing fat was dried at 100 °C, cooled in a desiccator, and reweighted. The oil yield of the olives was expressed as a percentage of dry weight (dw). For each sampling, three independent extractions were performed. Results calculated from triplicate data were expressed as means ± standard deviations.

### Biochemical analysis in olive pericarp

Chlorophyll and phenols compounds quantification was performed from the drupe pericarp. A pool of − 80 °C-stored drupes (*n* = 100) were dried, separately pulverized in liquid nitrogen, using a mortar and pestle, and lyophilized. For each sampling, three independent extractions were performed and for each replicate three measurements were carried out. Results calculated from triplicate data were expressed as means ± standard deviations.

#### Chlorophyll quantification

##### Total chlorophyll

extraction was carried out according to the method described previously [33] using 100 mg aliquot of the dry powder. Total amount of chlorophyll a and b was determined according to the method by [34] measuring Absorbance (A) A_646.8_ and A_663.2_ using a Cary 50 Bio (Varian, Turin, Italy) spectrophotometer.

#### Phenolic compunds quantification

##### Phenols compounds

were extracted as described in [33, 35], treating 200 mg of the dried powder in 15 ml of methanol/acetone (1:1), saturated with sodium disulfite and centrifugated at 5000 g at 4 °C, repeating the procedure for three times until we obtained a colourless pellet. The pooled supernatants were then dried under vacuum at 45 °C and the residue was dissolved in water (8 ml) and treated with hexane so that pigment and most of the lipids were removed. Finally, the phenol compounds were extracted by applying for six times ether/ethyl acetate (1:1) at a 1:1 solvent to water phase ratio. The extracts were dehydrated with anhydrous sodium sulphate, filtered, and dried under vacuum at 30 °C. The residue was dissolved in methanol (5 ml) and used for HPLC analysis, carried out according to [36] by HPLC apparatus (1100 Series, Agilent, Milan, Italy) with a photodiode array detector using a column inertSil ODS-3 (5 μm, 15 cm × 4.6 mm i.d.) equipped with a Spherisorb S5 ODS-2 (5 μm, 1 cm × 4.6 mm i.d., Sigma-Aldrich srl, Milano, Italy) precolumn. Operating conditions: elution with 0.2% acetic acid (pH = 3.1) and methanol, injection volume 20 μl; flow rate 1.5 ml/min, total run time 60 min. The solvent gradient changed as follows: initial composition was 95% acetic acid and 5% methanol, and the gradient changed as follows. The concentration of methanol was maintained for 2 min, then it was increased to 25% in 8 min, and finally, the methanol percentage was increased to 40, 50, and 100% in 10 min periods. It was maintained at 100% for 5 min. Initial conditions were reached in 15 min. Chromatograms were acquired at 280 and 240 nm. Individual phenols are expressed as mg per kg of dry weight olive pulp (mg/kg dw).

*Reference Compounds:* oleuropein, ligstroside, tyrosol (p-HPEA), hydroxytyrosol (3,4-DHPEA), elenolic acid dialdehyde linked to tyrosol (p-HPEA-EDA), elenolic acid dialdehyde linked to hydroxytyrosol (3,4-DHPEA-EDA), elenolic acid linked to tyrosol (p-HPEA-EA), elenolic acid linked to hydroxytyrosol (3,4-DHPEA-EA), were quantified by using the external standard method at 280 nm. Elenolic acid (EA) was detected at 240 nm.

#### Virgin olive oil analysis

After harvesting, drupes were immediately processed for oil extraction. Only healthy drupes, without any kind of infection or physical damage were processed, and the oils were obtained using an Oliomio – milling machine (Toscana Enologica Mori, Tavarnelle Val di Pesa, Firenze, Italy). For each sampling, a pool of drupes (10–15 kg) were cleaned from leaves and crushed with a hammer crusher. The obtained paste was mixed at room temperature for 30 min, centrifuged (1300 g for 3 min), and then transferred into dark glass bottles. The oils were stored at 4 °C until analysis.

#### VOO quality indices

Analytical methods described in the EUC/2568/91 EU Regulations and subsequent amendments and additions were used to estimate the free fatty acids (FAs), the peroxide value and the UV absorption characteristics at 232 nm and 270 nm (K_232_ and K_270_, respectively). Free acidity was given as a percentage of oleic acid and PV expressed in milliequivalents of active oxygen per kilogram of oil (mEquiv O_2_/kg). Spectrophotometric determinations were made using an UV − Vis 1800 instrument (Shimadzu Co., Kyoto, Japan). For each sample three independent measurements were carried out.

#### Analysis of free FA

The free FAs were prepared as described in the Commission Regulation No 2568/91 and N. 1429/92 of the EU and by the International Olive Council (2015). The chromatographic separation was carried out with an Agilent 6890 gas chromatograph (Agilent, Waldbronn, Germany), apparatus equipped with a fused-silica SP2340 Supelco column (60 m 0.25 mm i.d., film thickness 0.20 mm) and a flame ionization detector (FID), linked to a HP Chemstation integrator. Operating conditions: carrier gas was helium (purity 99.999%); flow rate 0.9 ml min^1^; injector temperature 260 °C; column was programmed from 150 °C (held for 7 min) to 230 °C at 3 °C/min (held for 15 min); FID temperature 260 °C. The results were expressed as relative percentages of the total area [37]. For each sample three independent analyses were carried out.

### Statistical analysis

Biochemical analysis data were compared on the basis of standard deviation of the mean values. Data were analysed using XLSTAT (Win v. 2016.3) to perform one-way-analysis of variance (ANOVA) at a 95% confidence level (*p* ≤ 0.05) to identify significant differences among all parameters analysed of VOO and drupes.

### RNA isolation

For each sampling, a pool of drupes (*n* = 500) stored at − 80 °C was pulverized in liquid nitrogen, using a mortar and pestle. Total RNA was isolated using 100 mg aliquot of powder through the RNeasy Plant Mini kit (Qiagen, Hilden, Germany) as previously described [38]. After DNase I (Roche, Milan Italy) treatment, total RNA was precipitated and finally re-suspended in RNase-free water. RNA was quantified by the NanoDrop Spectrophotometer ND-2000 and its integrity was checked by electrophoresis. For each sampling three independent RNA extraction were carried out. Only the samples with the following values were used both for cDNA library preparation and quantitative PCR: OD260/280 = 1.8~2.2, RNA 28S:18S ≥ 1.0, and RNA Integrity Number (RIN) ≥ 7.0. for each sampling three independent RNA extraction were carried out.

#### RNA-seq library synthesis

For each sampling, an aliquot (3 μg) of total RNA derived from one replicate extraction was used.

Cytoplasmatic rRNA removal was performed using the Ribo-Zero rRNA removal Kit (Epicentre, Madison, WI, USA) and rRNA-depleted RNA was used to prepare six RNA-seq libraries (10 masl GM, 10 masl TP, 200 masl GM, 200 masl TP, 700 masl GM and 700 masl TP) using the TruSeq Stranded Total RNA Sample Prep Kit (Illumina, San Diego, CA, USA), according to the manufacturer’s instructions. Libraries were sequenced on the Illumina HiSeq2000 platform at IGA Technology Services in Udine (Italy) and single-end reads of 50 bp were generated for each fragment.

#### sscDNA synthesis for quantitative PCR

For each sampling, an aliquot of total RNA (3 μg), derived from each of the three independent replicates, was interacted with SuperScript III Reverse Trascriptase and oligo dT(22) for cDNA synthesis, according to the manufacturer’s instructions (Invitrogen, Milan, Italy).

### Analysis of RNA-Seq reads

RNA-Seq reads in FASTQ format were inspected using FASTQC program (http://www.bioinformatics.babraham.ac.uk/projects/fastqc/) while adaptors and low quality regions (phred cut-off 20) were trimmed using TrimGalore (http://www.bioinformatics.babraham.ac.uk/projects/trim_galore/), excluding reads with final length less than 30 bases.

Cleaned reads were subsequently aligned onto the recently released olive wild genome (v1.0) [29] by means of STAR [39] (using as parameters: --outSAMtype BAM SortedByCoordinate --outSAMattributes All --outFilterMultimapNmax 1).

The generalized fold change algorithm (GFOLD) [40], was used to assign reads to known olive annotations and perform differential gene expression. Olive wild genome v1.0 in fasta format and annotations in gff3 format were downloaded from Phytozome v13 [41].

### Quantification of olive transcripts

Read counts per transcript were converted in RPKM values according to [42], by applying the GFOLD robust algorithm [40]. Comparison of gene expression was performed calculating log_2_ of fold-changes using custom python scripts.

### Quantitative real-time PCR (qRT-PCR)

To validate transcriptomic data, the expression level of selected genes was analysed also through qRT-PCR analysis using a STEP ONE instrument (Applied Biosytems). Primers described and tested by [20, 23, 38, 43−45] were used for qRT-PCR analysis as reported in Additional file 2: Table S1. Amplification reactions were prepared in a final volume of 20 μl by using the sscDNA (25 ng) as template as described in [46, 47]. After the reaction, the existence of an unique PCR product was confirmed by evaluating the ‘melting curve’ [48] through an increase of 0.5 °C every 10 s, from 60 °C to 95 °C. The results were analysed using STEP One Software 2.0 (Applied Biosystems), according to the 2^-ΔΔCT^ method [49]. For each sampling three independent amplifications and three replicates for each amplification were performed. Results calculated from replicate data were expressed as means ± standard deviations. Part of the results of qRT-PCR analysis are reported as Additional file 8: Figure S6.

### Metabolic pathways analysis

Metabolic pathway analysis was analysed by using Interactive Pathways (ipath) (version 2.0) (http://pathways.embl.de/), according to [50]. Through GO (Gene Orthology) id, the expression of a specific gene family was summed from all family members encoding the gene. Metabolic pathways related with lipid and phenol metabolism were produced manually. To understand the dynamic changes and absolute expression magnitude during fruit maturation, two different colours were applied to indicate different RPKM values of Genes.

Fatty acids and phenols-related pathways analysis was performed by using the online tool KEGG Mapper (https://www.genome.jp/kegg/mapper.html). This Web-based system allows the final user to explore large-scale gene expression data-set and mapping the genes identified by the transcriptomic analysis by using KEGG identifiers present in the target database.

## Results and discussion

### Metabolic characterization of drupes

Preliminarly, for each population (i.e. 10, 200, 700 masl) some drupes randomly collected at 150 and 180 DAF (*n* = 100 for each DAF and for each population) were analysed to define the Ripening Index (RI) (Table 1). It was observed that for all the populations, the samples collected after 150 and 180 DAF corresponded to the green mature stage (RI = ranging from 0.0 to 0.5 in drupes from different masl) and to the turning purple stage (RI = ranging from 2.6 to 3.2 in drupes from different masl), respectively [32]. From here on, we will only refer to the drupe maturation stage as GM (green mature) and TP (turning purple) stages.

**Table 1:** Ripening index (RI), dry matter, chlorophyll content (Chl), chlorophyll retention (R) during ripening were estimated in green mature (GM) and turning purple (TP) drupes of ‘Carolea’ populations growing at different meters above sea levels (masl)

Population	stage	RI	Dry matter (% FW)	Chl *a (mg/g DW)*	Chl *b (mg/g DW)*	Chl (*b/a*)	Chl (*a+b*) *(mg/g DW)*	R
10 masl	GM	0.5^b^	46.6 ± 1.1^b^	0.68 ± 0.08^a^	0.38 ± 0.05^c^	0.56^d^	1.06 ± 0.06^b^	-
	TP	3.2^a^	54.3 ± 1.5^a^	0.17 ± 0.02^b^	0.36 ± 0.04^c^	2.10^b^	0.53 ± 0.03^c^	50^ns^
200 masl	GM	0.0^b^	46.0 ± 0.9^b^	0.70 ± 0.08^a^	1.13 ± 0.08^a^	1.62^c^	1.83 ± 0.08^a^	-
	TP	2.6^a^	55.6 ± 1.8^a^	0.20 ± 0.02^b^	0.64 ± 0.06^b^	3.14^a^	0.84 ± 0.05^b^	46^ns^
700 masl	GM	0.0^b^	46.1 ± 1.0^b^	0.55 ± 0.06^a^	0.82 ± 0.07^b^	1.47^c^	1.37 ± 0.06^a^	-
	TP	2.7^a^	56.5 ± 1.9^a^	0.23 ± 0.02^b^	0.50 ± 0.03^c^	2.19^b^	0.72 ± 0.03^b^	53^ns^

Data are expressed as means ± standard errors. Significant differences between means are shown by different letters (*P*≤0.05) (Student’s t-test); *ns* not significant, *DW* Dry weight, *FW* Fresh weight

**Fig. 1:**
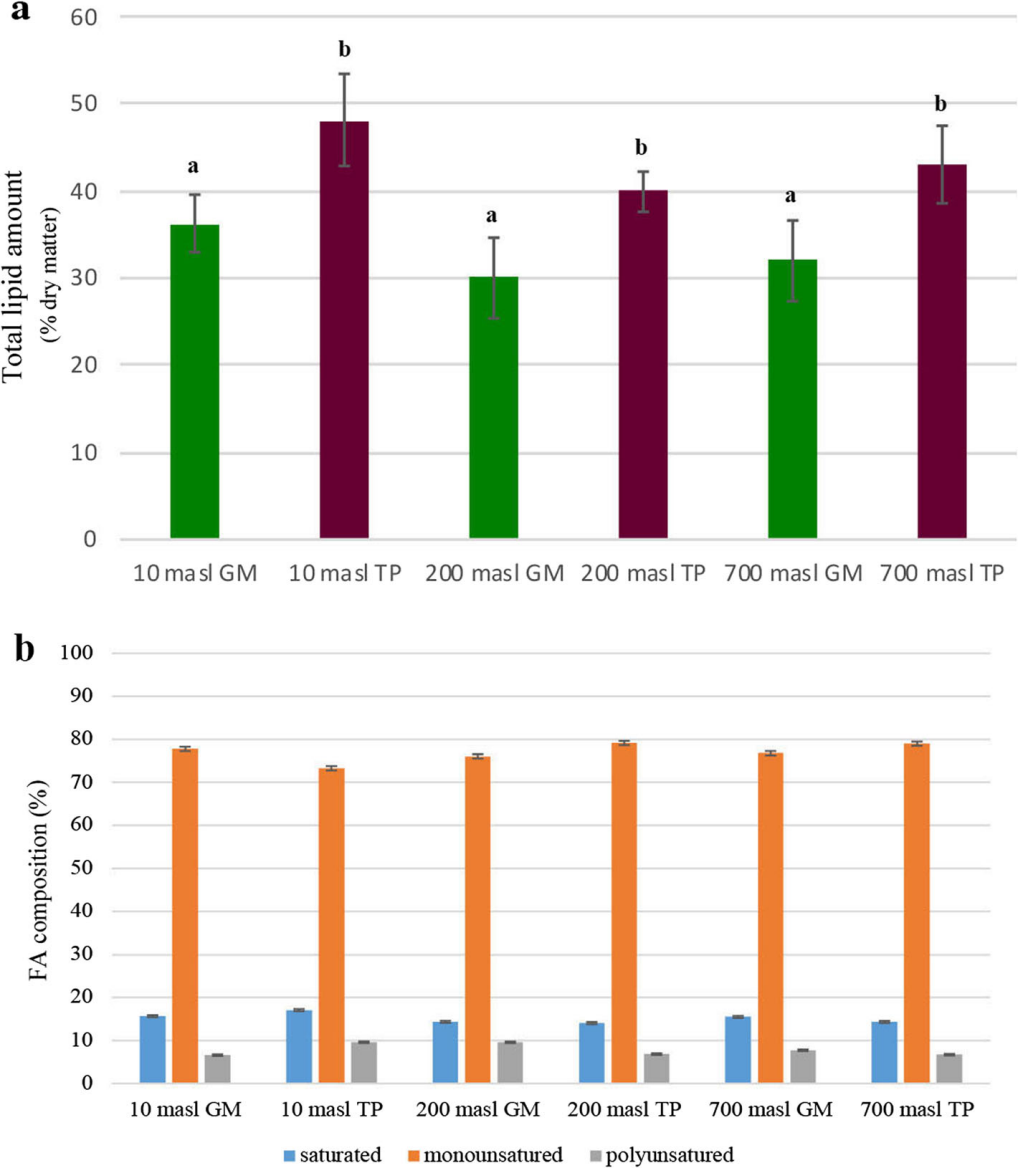
**a** Lipid content in the pericarp (% dry matter) and **b** oil fatty acids composition, expressed as percentages of saturated, monounsatured and polyunsatured FAs (% total FAs content), estimated in green mature (GM) and turning purple (TP) drupes of ‘Carolea’ populations growing at different meters above sea levels (masl). The results were reported as mean values (± standard deviation) of three replicates. Significant differences are shown by different letters (*P* ≤ 0.001) (Students t-test)

**Fig. 2:**
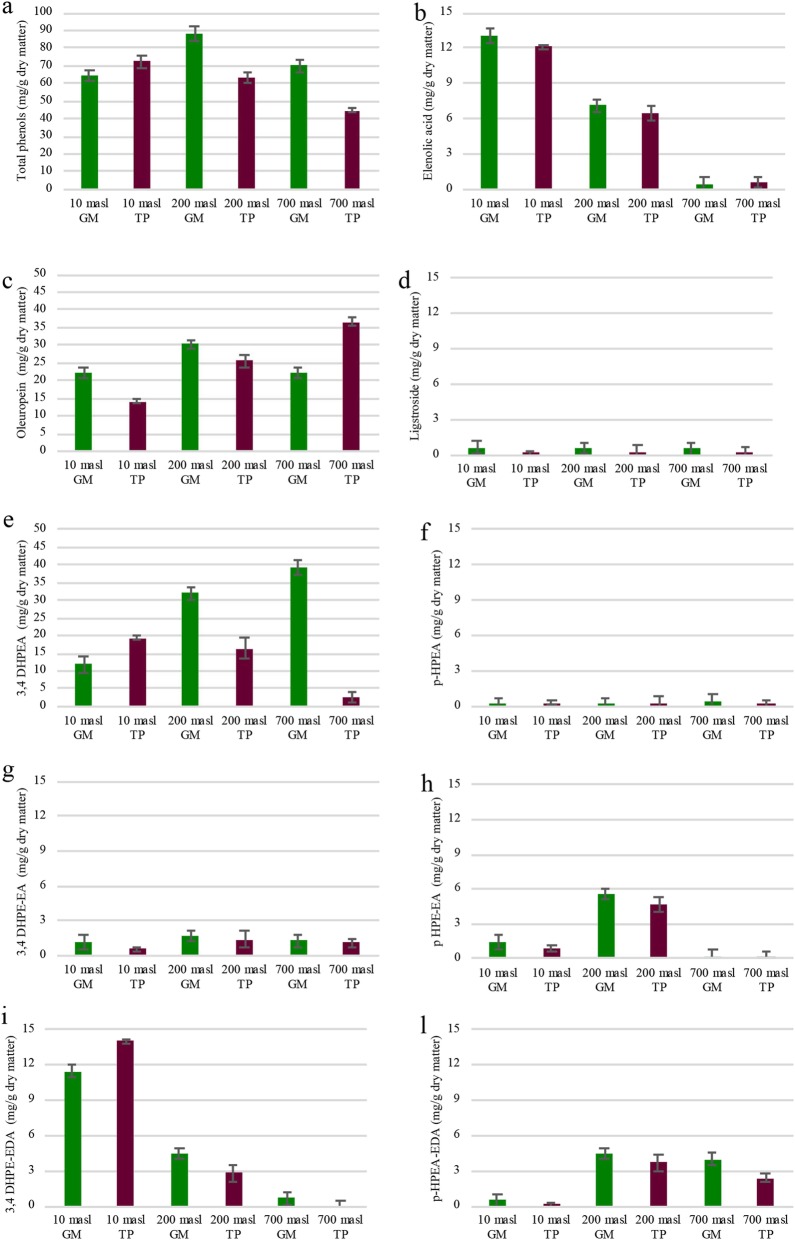
Total and specific phenolic content, estimated through HPLC analysis and expressed as mg per Kg of dry matter, in green mature (GM) and turning purple (TP) drupes of ‘Carolea’ populations growing at different meters above sea levels (masl). **a** Total phenols, **b** Elenolic Acid (EA), **c** hydroxytirosol (3–4DHPEA), **d**  tyrosol (p-HPEA), **e** elenolic acid linked to tyrosol (p-HPEA-EA), **f** elenolic acid linked to hydroxytyrosol (3,4-DHPEA-EA), **g** elenolic acid dialdehyde linked to tyrosol (p-HPEA-EDA), **h** elenolic acid dialdehyde linked to hydroxytyrosol (3,4-DHPEA-EDA). The results were reported as mean values (± standard deviation) of three replicates

To further characterize the drupe metabolic state, some relevant parameters such as the chlorophyll content (Table 1), the level and composition of lipids (Fig. 1 and Additional file 3: Table S2) and phenolics (Fig. 2) were also evaluated.

A significant decrease of total chlorophylls amount was observed in drupes at TP vs GM stage whatever population was considered (Table 1). This result is consistent with the RI detected at TP stage, indicating that drupe at this stage were characterized by an incomplete purple pigmentation of the fruit surface due to the simultaneous anthocianins accumulation and chlorophyll degradation [51]. Moreover, the relative rate of chlorophylls disappearance (R) was quite comparable in the three populations. However, and very interestingly, when chlorophyll *b/a* ratio was analysed clear differences between populations could be observed. Namely, at GM stage such ratio was significantly lower in the population at 10 masl compared with those at 200 and 700 masl which showed similar values. An increase of chlorophyll *b/a* ratio during ripening occurred in all the populations, but at different extent being about of 2.8, 0.9, 0.5 fold in populations at 10, 200, 700 masl, respectively. As a result, at the TP stage the highest ratio was observed in the population at 200 masl (Table 1).

The chlorophyll contents, and in many fruits the chlorophyll *b/a* ratio, can vary in relation to the genus, species, variety, environmental factors, and ripeness stage [52, 53]. In our case, differences were found at the intra-varietal level, which imply that in the cultivar Carolea the catabolism of chlorophylls during ripening as well as the structure of the photosynthetic apparatus clearly differ in relation the different environmental conditions impending at the different masl.

Concerning lipid components, in all the populations an increase of about 30% was detected in the total amount, estimated as a percentage on dry matter, when comparing drupes at GM vs TP stage (Fig. 1a). Moreover, although differences did not appear statistically significant, the highest and the lowest levels were detected in the populations at 10 and 200 masl respectively (Fig. 1a). The FAs composition of the oils derived by processed drupes was also analysed and summarized in the Additional file 3: Table S2. According to data in literature [54, 55] in all the populations the highest fraction was related to monounsaturated FAs (Fig. 1b). However, statistical analysis evidenced that in the samples from 200 and 10 masl the balance between saturated and unsaturated FAs slightly changed during the transition to TP stage, while it remained unvaried in the samples from 700 masl (Fig. 1b). In particular we observed that in the samples from 200 masl the percentages of monounsaturated and polyunsaturated FAs increased and decreased respectively, while an opposite pattern was reported in the samples from 10 masl (Fig. 1b). Therefore, some significant differences in FAs composition resulted to occur in relation to both the developmental stage and masl. Nevertheless, in all the samples the ratio oleic acid/linoleic acid was greater than 7 (Additional file 3: Table S2) according to the standard of a high quality oil [56]. Moreover, the parameters related to oil quality, such as the free acidity value (FA), the primary oxidation value (PV), dienes/trienes conjugation index, carbonylic compounds presence (Additional file 3: Table S2) felt within the accepted values for extra virgin olive oils (EVOOs) (EEC, 1991; IOC 2006). Therefore, the oil obtained from all the samples globally featured of high quality. That is consistent with data showing that in Carolea cultivar the TP stage corresponds to the ripeness stage in which the chemical and sensorial characteristics of olive oil are optimal [57, 58].

The amount of specific phenolics whose putative pathway is reported in the Additional file 4: Figure S2, was also evaluated (Fig. 2). Attention was largely focused on the secoiridoids group and their derivatives, as relevant players in conferring both sensorial and healthy characteristic to olive products (Fig. 2). These components, derive from iridoids, are present as oleosides and include oleuropein, ligstroside and their derivatives (Additional file 4: Figure S2). Their abundance is related to enzymatic activities present in olive fruits which have not yet been fully clarified.

Until now, it is known that oleuropein is the most abundant fraction [59] and its amount depends on the activity of endogenous ß-glycosidase enzyme which hydrolises it into glucose and oleuropein aglycon. This latter compound can be further esterolysed to produces hydroxytyrosol, which is considered one of the most bioactive phenol molecules. On the other hand, the aglycon structure can also undergo to several chemical rearrangements, such as decarboxylation, methylation, or oxidation, leading to the formation of new phenol aglycon structures, including unstable ketoenolic tautomer forms (e.g., mono- and dialdehydic forms) [60−61].

Some differences in relation to both the masl and the developmental stage were observed when considering the total amount of the selected phenolics (Phs). Indeed, at the GM stage the highest Phs content was detected in the drupes of populations growing at 200 masl. Moreover, and more interestingly, during the transition from the GM to TP stage, Phs amount significantly decreased in the drupes of populations growing at 200 and 700 masl, whereas increased in the drupes of populations growing at 10 masl (Fig. 2a). This opposite behaviour resulted in a highest Phs content in the TP drupes of populations growing at 10 masl as compared to those of other two populations. More clear differences at both quantitative and temporal level were observed taking into account singularly the level of each phenol. In particular, it was evident that in all the samples the major fraction was related to oleuropein, and hydroxytirosol (3–4-DHPEA) (Fig. 2c, e). However, taking into account singularly the level of each phenol it could be observed clear quantitative and temporal differences in the accumulation pattern of secoiridoids and related compounds. Among these, a very low and stable amount of EA was observed in the drupes of population growing at 700 masl compared to those at 200 and 10 masl. Moreover, at the GM stage oleuropein content was significantly higher in the drupes of populations growing at 200 masl compared with those of populations growing at 10 and 700 masl. In addition, according to data in literature [21] in the drupes of populations growing at 10 and 200 masl the amount of such compound decreased of about 1/1.5 fold during the transition to the TP stage. By contrast in the drupes of population growing at 700 masl oleuropein content underwent a 1.5 fold increase (Fig. 2c). On the other hand, a higher level of dialdeid oleuropein aglycon (3–4-DHPEA-EDA), was also observed during the transition to TP stage, in the populations growing at 10 masl (Fig. 2i). By contrast dialdeid ligustroside aglycon (p-HPEA-EDA) amount was higher in the GM drupes from 200 and 700 masl than in the drupes from 10 masl (Fig. 2l).

### Comparative transcriptome analysis

Comparative transcriptomic analysis was carried out on Carolea drupes at different climatic conditions using deep transcriptome sequencing through the Illumina HiSeq2000 platform. Six libraries (10 masl GM, 10 masl TP, 200 masl GM, 200 masl TP, 700 masl GM and 700 masl TP) were prepared for our purposes. Each library produced on average 42 million single end reads and about 95% of these were used for further analyses, after an appropriate cleaning procedure (see Material and Method section).

After transcript quantification and genome mapping, differentially expressed genes (DEG) between TP vs MG drupes were identified (see Material and Method section) [45].

**Fig. 3:**
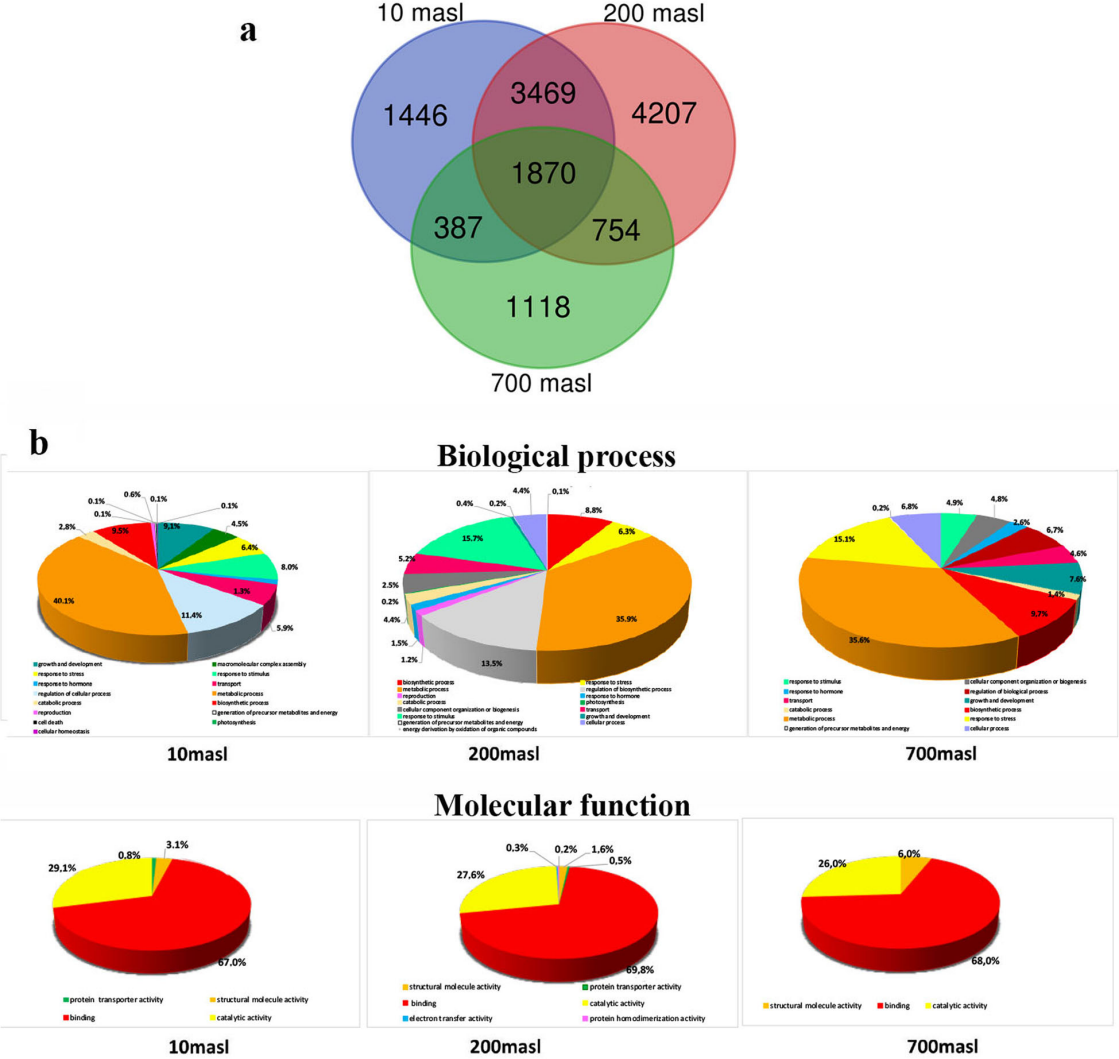
**a** Venn diagram and **b** classification of differentially expressed genes (DEGs) according to (up) biological process and molecular function (down) in turning purple (TP) vs mature green (GM) drupes of ‘Carolea’ populations growing at different meters above sea level (masl)

As shown in a Venn diagram (Fig. 3a), libraries from drupes of populations growing at 10 and 200 masl shared a higher number of DEGs compared to the library from drupes of population growing at 700 masl, while only 1870 DEGs were commonly expressed in all the three libraries (Fig. 3a). This result suggests that during drupe ripening gene expression is differentially modulated at the different masl with the largest differences being observed in samples from 700 masl.

Based on functional annotation, for each library, the DEGs were classified into two gene ontology categories: biological process and molecular function (Fig. 3b). A general observation is that, despite the information derived from each population was quantitatively different, the spectrum of categorized functions and processes is quite comparable. In particular, concerning the molecular function category (Fig. 3b), in all the comparisons DEGs were categorized in large proportion as involved in binding (from 67 to 70%) and catalytic activity (from 26 to 29%). Whereas, in the biological process-based categorization (Fig. 3b) DEGs related to metabolic processes represented the major fraction in all the three populations, ranging from 35 to 40% (Fig. 3b). These results are consistent with a still active metabolism of drupes throughout the transition from GM to TP stage. Growth and development categories were also present for both drupes of ‘Carolea’ growing at 10 masl (4%) and 700masl (8%) and almost absent in those of plants growing at 200 masl, suggesting a more accelerated arrest of these processes in the latter. In addition, only in drupes of plants growing at 10 masl, a fraction of DEGs (11%) was categorized as regulation of cellular process. Differences were also detected concerning stimulus response category which exhibited the highest fraction in drupes of plants growing at 200 masl (16%) as compared to those at 10 masl (8%) and 700 masl (5%). Finally stress-related genes covered a relevant fraction of DEGs (15%) in the drupes of ‘Carolea’ growing at 700 masl, higher than in those at 200 and 10 masl (6%) (Fig. 3b), suggesting that at the higher altitude the drupes are exposed during phase transition to major stressful condition compared to those growing at 200 and 10 masl. Based on the moderate thermophily [23] of olive plants, we hypothesize that the lower temperature impending on drupes at 700 vs 200 and 10 masl (Additional file 1: Figure S1a) could account for this differential response.

### Maturation-related variations of metabolic pathways at the different masl

To obtain a global view of drupe metabolism during the transition from GM to TP stages and verify whether and how it differs in relation to the altitude, KEGG pathway mapping tool (https://www.genome.jp/kegg/tool/map_pathway2.html) was used. The DEGs selected from the RNA-Seq transcriptomic data where searched against the KEGG pathway maps to create interactive metabolic networks [62, 63] (Additional file 5: Figures S3, Additional file 6: Figures S4, Additional file 7: Figures S5).

It was evident that during the transition from GM to TP stages there was a prevalent gene downregulation in the drupes of all the three populations even if more pronounced in those growing at 10 masl compared to both 200 and 700 masl (Additional file 5: Figures S3, Additional file 6: Figures S4, Additional file 7: Figures S5). Notwithstanding, in all the populations some gene upregulation could be observed dealing with genes involved into energetic metabolism (i.e pyruvate metabolism, Krebs cycle), carbohydrate metabolism and amino acids catabolism as well as into lipid metabolism, this latter limited to the populations growing at 200 and 700 masl (Additional file 5: Figures S3, Additional file 6: Figures S4, Additional file 7: Figures S5). In addition, some genes involved in flavonoid and phenylpropanoids biosynthesis were also upregulated in the drupes of populations growing at 10 and 200 masl, respectively (Additional file 5: Figures S3, Additional file 6: Figures S4).

Although at posttranscriptional level a different regulation can occur, this transcriptional pattern, highlighted that during the transition from GM to TP stages both lipid- and phenol-related genetic pathways are differentially modulated in the drupes growing at different masl. This result is consistent with the differences detected both in lipid amount and composition (Fig. 1) and in the amount of the different analysed phenolic compounds (Fig. 2) as well as in the RI value (Table 1) related to drupe veraison occuring at TP stage, due to anthocyanins accumulation [51, 64]. Notably, according to transcriptomic data showing a higher or more prolonged activation of the genetic pathway related to anthocyanins production in the drupes of plants growing at 10 masl, these exhibited a higher RI as compared to those of plants growing at 200 and 700 masl.

Based on this interactive network, we moved to analyse in more details the differential expression of genes related to lipids and phenols metabolic pathways, due to their close relationship with fruit quality.

### Expression pattern of genes related to the lipid metabolism

**Fig. 4:**
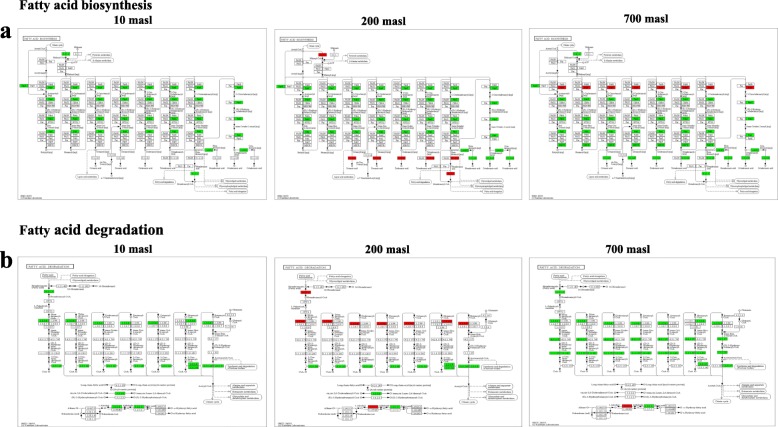
Genes differentially expressed (DEGs) along the pathway of fatty acid biosynthesis and degradation in in turning purple (TP) vs mature green (GM) drupes of ‘Carolea’ populations growing at different meters above sea level (masl). identified through a transcriptomic approach

**Fig. 5:**
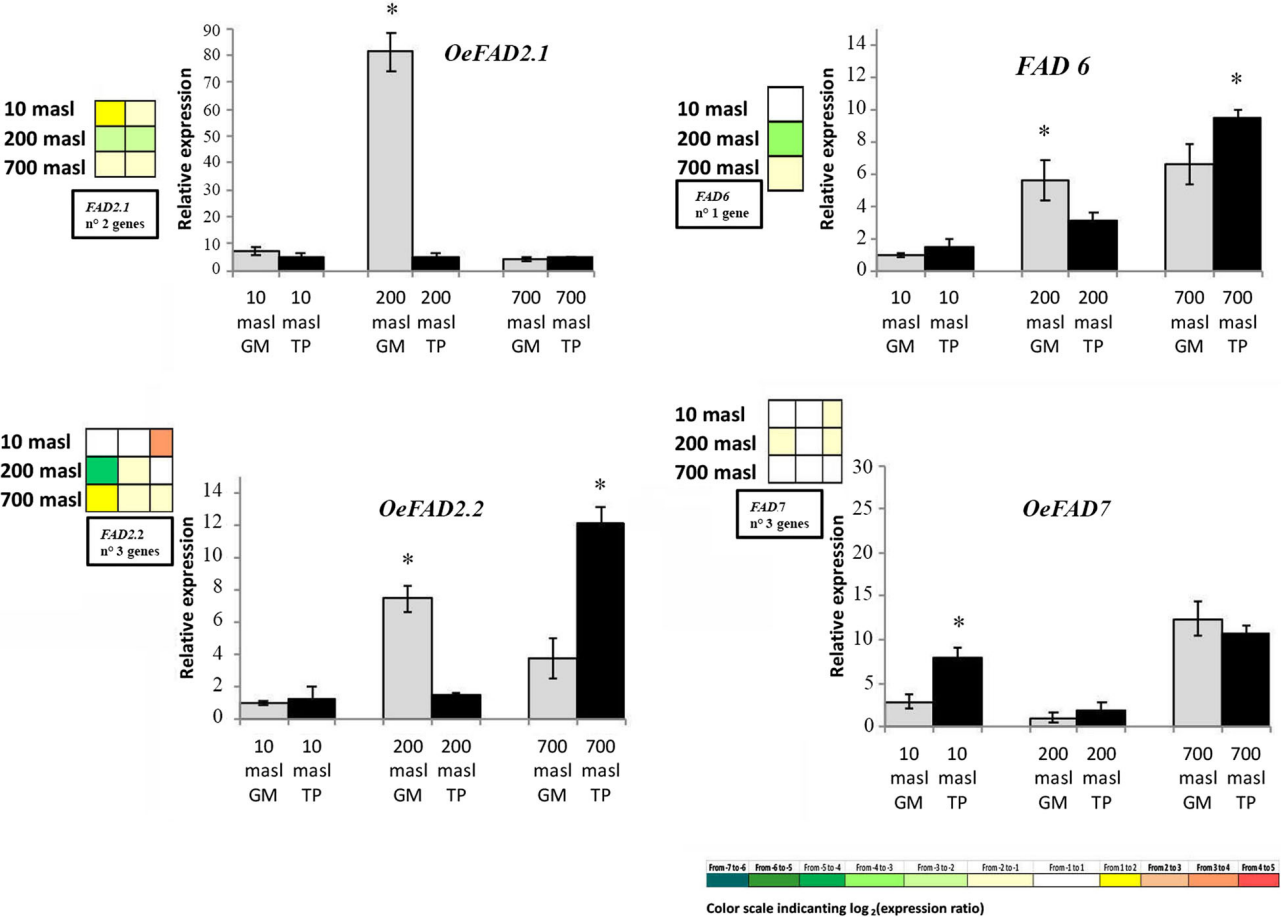
Relative expression levels of FAD genes in green mature (GM) and turning purple (TP) drupes of ‘Carolea’ populations growing at different meters above sea level (masl), estimated by qRT-PCR after normalization with the *CRYPTOCHROME2* (*CRY2*) housekeeping gene. The results were reported as mean value (± standard deviation) of three replicates. Asterisks indicate significant pairwise differences using Student’s t-test (*P* ≤ 0.05). The grid at the left of each graph shows the gene expression pattern detected in the drupes through transcriptomic analysis

Concerning lipid metabolism, attention was in particular paid to the biosynthesis and degradation of FAs (Fig. 4). Despite a general downregulation of FAs biosynthesis in all the samples, an upregulation of specific genes along the pathway was observed only in the drupes of populations growing at 200 and 700 masl (Fig. 4a). A downregulation of metabolic pathway related to FAs degradation was also observed in the drupes of populations growing at 10 and 700 masl, even if more extensive in the latter as compared to the former (Fig. 4b). By contrast, in the drupes of population growing at 200 masl an equilibrate ratio between up- and downregulated DEGs along this degradation pathway was observed (Fig. 4b). Attention was then paid to the expression level of *FAD* desaturation genes (*FADs*) specifically involved in the biosynthetic pathway of unsatured FAs which strongly impact on oil quality [23]. The expression level of these genes was estimated also through qRT-PCR analysis to validate sequencing data. Both transcriptomic and qRT-PCR analysis, evidenced that at 700 masl *OeFAD2.2* and *FAD6* genes were upregulated in TP vs GM (Fig. 5). An opposite behaviour was observed in samples from 200 masl where *FADs* expression was globally downregulated (Fig. 5). Finally, in the populations growing at 10 masl, *FADs* expression was globally lower compared to samples from 200 and 700 masl and only *OeFAD7* appeared to be upregulated in TP vs GM stages (Fig. 5).

This differential expression pattern of *FADs* detected between drupes from different maturation stage and populations is globally consistent with a large body of evidence showing that the expression of these genes is spatially and temporally regulated during olive fruit ripening and can be also modulated by external factors, first of all by temperature, independently by genotype  [13, 23, 65]. In particular cold-induced expression of *OeFAD7* has been reported in different olive cultivar [23] and could account for the highest level of *OeFAD7* in samples from 700 masl as compared to samples from 200 and 700 masl. Globally these results support the idea that at 700masl a high stability of FAs is in place.

### Expression pattern of genes related to the phenols metabolism

**Fig. 6:**
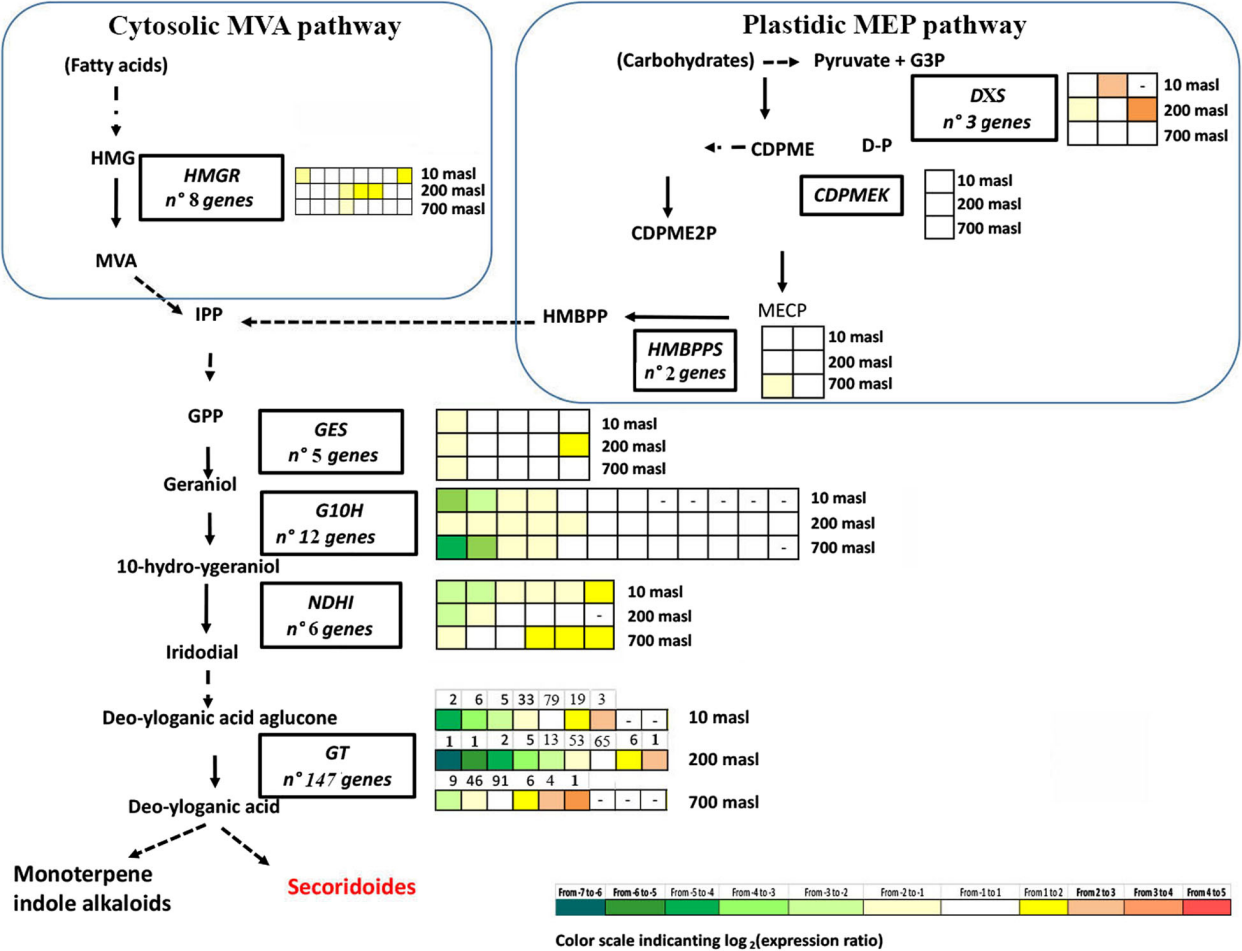
Partial scheme of phenol biosynthetic pathway. Genes differentially expressed (DEGs) along the pathway in turning purple (TP) vs mature green (GM) drupes of ‘Carolea’ populations growing at different meters above sea level (masl).are showed in the boxes. The grids on the left of each box indicate the expression pattern detected through transcriptomic analysis in the drupes of ‘Carolea’ populations growing at different meters above sea level (masl)

**Fig. 7:**
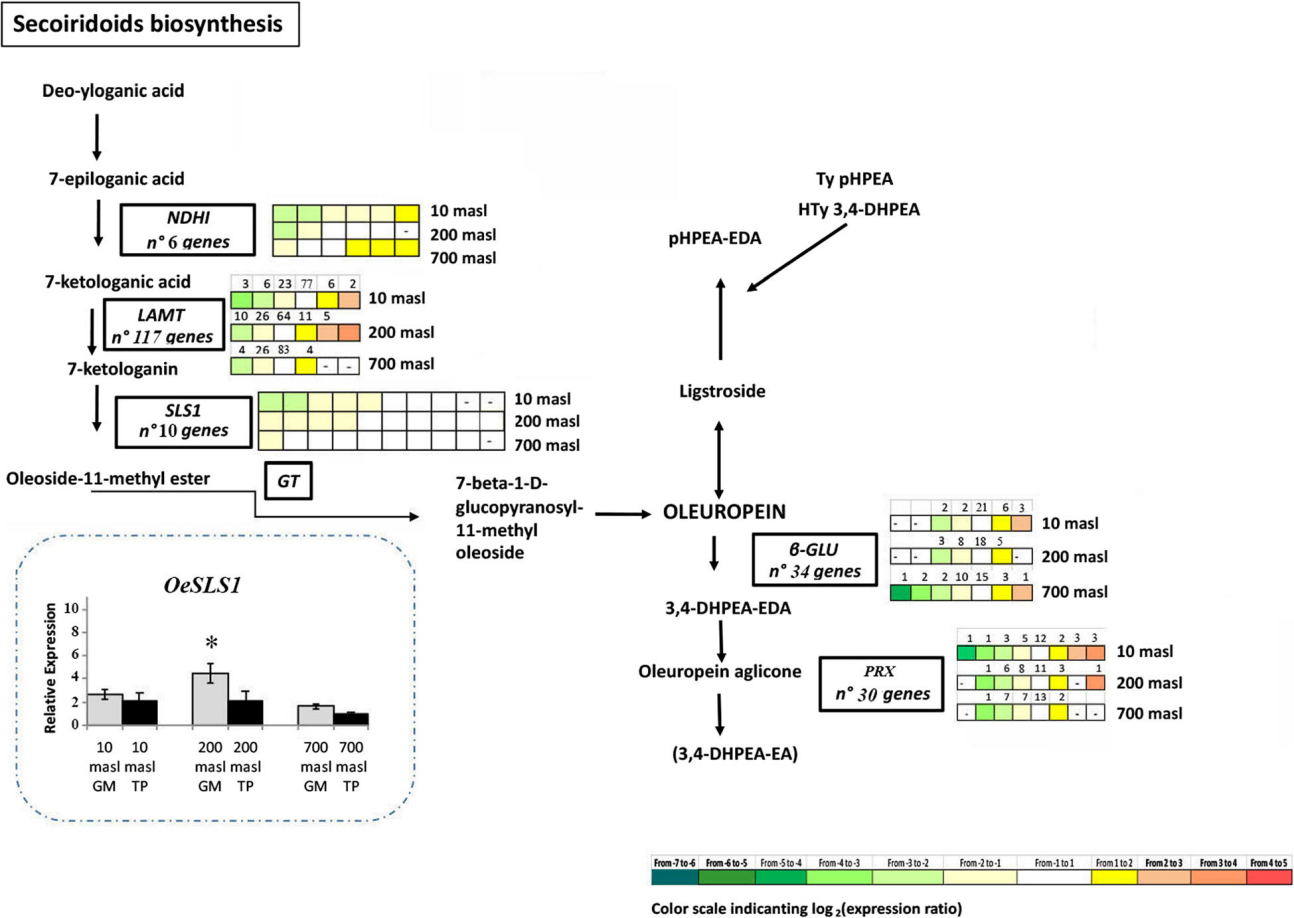
Scheme of putative secoridoids biosynthetic pathway. Genes differential expressed (DEGs) along the pathway in turning purple (TP) vs mature green (GM) drupes of ‘Carolea’ populations growing at different meters above sea level (masl) are showed in the boxes. The grids on the left of each boxes indicate the expression pattern detected through transcriptomic analysis in the drupe of ‘Carolea’ populations growing at different meters above sea level (masl). In dashed box the relative expression levels of SLS1 genes in green mature (GM) and turning purple (TP) drupes of ‘Carolea’ populations growing at different meters above sea level (masl), estimated by qRT-PCR after normalization with the *ELONGATOR FACTOR1* alpha (*EF1*) housekeeping gene. The results were reported as mean values (± standard deviation) of three replicates. Asterisks indicate significant pairwise differences using Student’s t-test (*P* ≤ 0.05)

**Fig. 8:**
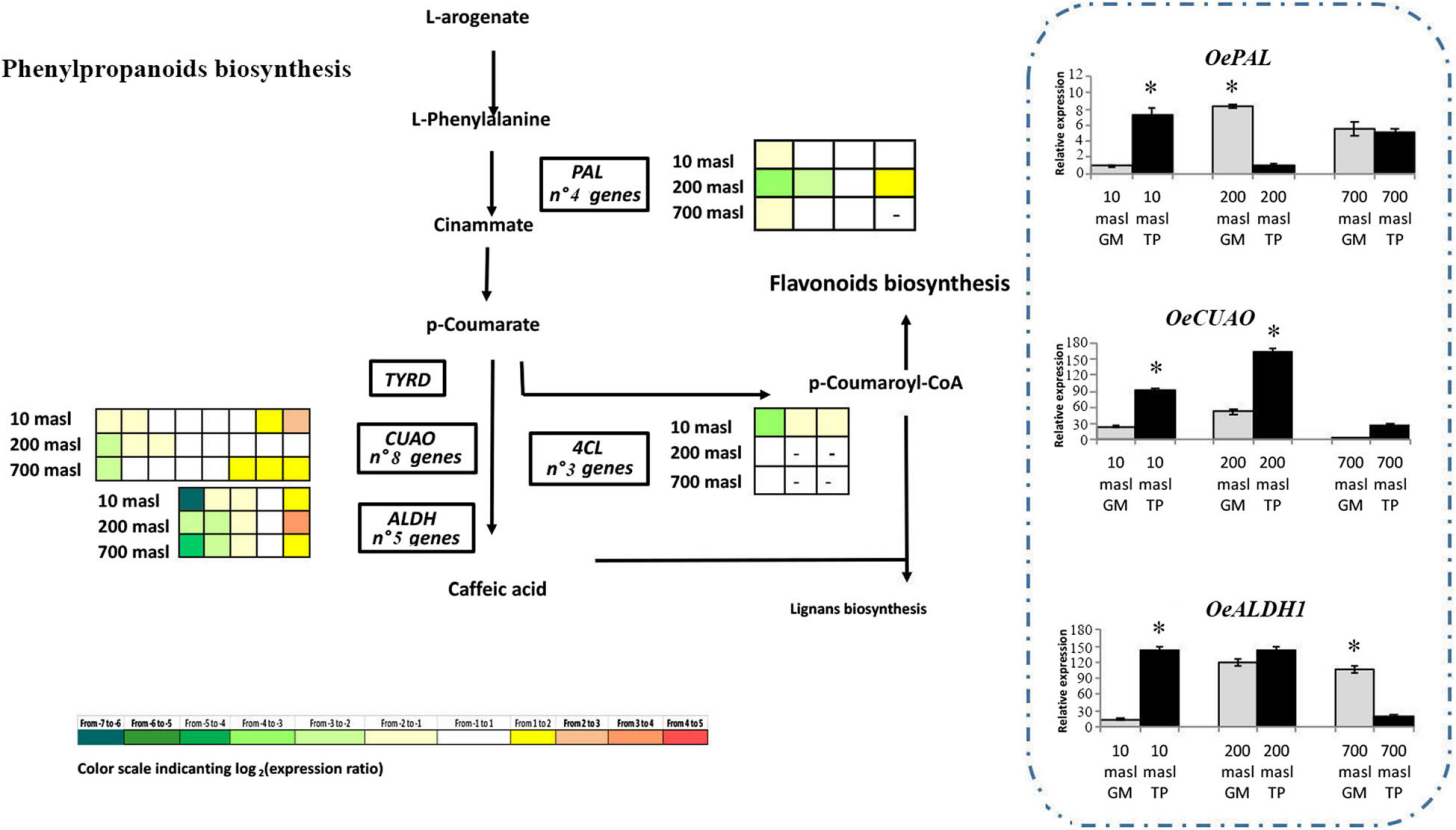
Simplified scheme of phenolpropanoid biosynthetic pathway. Genes differential expressed (DEGs) along the pathway in turning purple (TP) vs mature green (GM) drupes of ‘Carolea’ populations growing at different meters above sea level (masl) are showed in the boxes. The grids on the left of each box indicate the expression pattern detected through transcriptomic analysis in the drupe of ‘Carolea’ populations growing at different meters above sea level (masl). In dashed box the relative expression levels of PAL, CUAO and ALDH genes in green mature (GM) and turning purple (TP) drupes of ‘Carolea’ populations growing at different meters above sea level (masl), estimated by qRT-PCR after normalization with the *ELONGATOR FACTOR1* alpha (*EF1*) housekeeping gene. The results were reported as mean values (± standard deviation) of three replicates. Asterisks indicate significant pairwise differences using Student’s t-test (*P* ≤ 0.05)

**Fig. 9:**
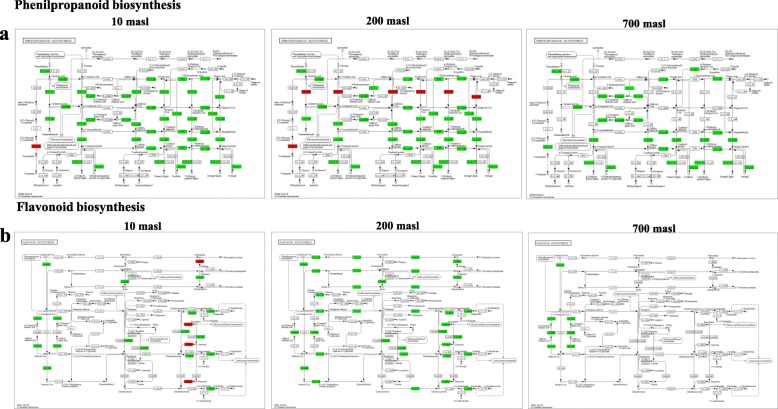
Genes differentially expressed (DEGs) along the pathway of phenylpropanoid and flavonoid biosynthesis in turning purple (TP) vs mature green (GM) drupes of ‘Carolea’ populations growing at different meters above sea level (masl), identified through a transcriptomic approach

Concerning phenolic compounds, the expression pattern of genes identified along the pathway is illustrated in Figs. 6, 7, 8 and 9. The expression level of some of these genes, analysed by qRT-PCR as validation of RNAseq data, is also reported (Figs. 7 and 8 dashed boxes). A general downregulation of the genetic pathway was observed during the transition from GM to TP stages. Notwithstanding, some specific genes resulted upregulated, mainly in drupes of populations growing at 200 and 10 masl as compared to that of drupes of populations growing at 700 masl (Figs. 6, 7, 8 and 9). Globally, these results indicated that starting from the plastidic methylerythritol 4-phosphate (MEP) and mevalonate (MVA) pathways and along all the branches of the pathway, the identified genes were differentially modulated when comparing the three populations.

An intriguing aspect of this differential expression pattern is related to the behaviour of genes involved in secoiridoids compounds (Fig. 7). Globally we observed that in the drupes of populations growing at 10 and mainly at 200 masl most genes involved in both biosynthesis (*NDHI, LAMT* and *SLS1*) and degradation (ß-*GLUs* and *PRX*) of oleuropein were upregulated in TP vs GM stages. By contrast, in the drupes of populations growing at 700 masl, some upregulation of biosynthesis-related genes was associated to a downregulation of catabolism-related genes (Fig. 7)*.* Although we cannot directly relate gene expression with compounds amount or activity, these results allow us to hypothesize that at the highest masl the degradation of oleuropein is reduced or delayed, as supported by its higher amount in TP drupes growing at 700 masl compared to those growing at 10 and 200 masl (Fig. 2c). Oleuropein is known for the beneficial healthy effects related to its high antioxidant activity. Therefore, the higher oleuropein content detected in TP drupes collected at 700 masl compared to those at 200 and 10 masl is consistent with the best quality conventionally assigned to oil produced by fruits produced by plant growing at low temperature.

## Conclusion

In conclusion, the present study, provides some interesting information on the modulation of gene expression in the fruit of ‘Carolea’ olive plants in relation to both the transition from green mature to turning-purple stage and the altitude of cultivation area. Analysis has been focused in major detail to lipid and phenol metabolism and the obtained results allow us to propose that at low temperature, such as that experienced by drupe growing at 700 masl, the modulation of gene expression during stage transition lead to promote fatty acids stability, while the milder temperatures impending at lower masl is involved in prolonging phenol biosynthesis. This result could be related to the well documented role of phenols in protecting plant and fruit against high irradiance and oxidative stress which are likely to largely occur during summer at low masl.

Finally, and very interestingly, in the drupes of ‘Carolea’ plants growing at 700 masl, gene expression was modulated so that a higher stability of oleuropein fraction was promoted compared to those growing at 10 and 200 masl, which resulted in a higher level of this secoiridoid at the TP stage, despite a global lower total Phs content. Note that oleuropein is tightly associated to the healthy properties of olive products and it is also largely known that oleuropein content dramatically decreases during drupe development and ripening [20, 36]. Therefore, the enhancement of oleuropein stability and content detected in TP drupes of cv Carolea plants growing at the higher masl represents a relevant trait for olive breeding program. Globally, this information could be of interest for biotechnological programs addressed to improve the quality and the health properties of olive products.

## Additional files

Additional file 1: **Figure S1. a** average temperature and **b** average precipitation level in the period from May to November 2012 recorded at 10, 200 and 700 masl sites. These informations were obtained by consulting historical databases available on the web (http://www.meteoam.it). (TIF 3637 kb)

Additional file 2: **Table S1.** List of the primers utilized for qRT-PCR analysis. (PPTX 41 kb)

Additional file 3: **Table S2.** Analytical parameters of oil derived from green mature (GM) and turning purple (TP) drupes of ‘Carolea’ populations growing at different meters above sea levels (masl). Significant differences are shown by different letters (*P* ≤ 0.05) (Students t-test). (PPTX 45 kb)

Additional file 4: **Figure S2.** Scheme illustrating the putative biosynthetic pathways of main phenols of olive fruit. (JPG 164 kb)

Additional file 5: **Figure S3.** Interactive pathways analysis during drupe maturation of ‘Carolea’ population growing at 10 masl. The red and blue lines indicate the up and down regulated pathways respectively. (PPTX 920 kb)

Additional file 6: **Figure S4.** Interactive pathways analysis during drupe maturation of ‘Carolea’ population growing at 200 masl. The red and blue lines indicate the up and down regulated pathways respectively. (PPTX 935 kb)

Additional file 7: **Figure S5.** Interactive pathways analysis during drupe maturation of ‘Carolea’ population growing at 700 masl. The red and blue lines indicate the up and down regulated pathways respectively. (PPTX 883 kb)

Additional file 8: **Figure S6.** Relative expression levels of *OeDHN*, *OeARF, OeGGH* and *OeH3* genes in green mature (GM) and turning purple (TP) drupes of ‘Carolea’ populations growing at different meters above sea level (masl), estimated by qRT-PCR after normalization with the *ELONGATOR FACTOR 1* alpha (*EF1*) housekeeping gene. The results were reported as mean values (± standard deviation) of three replicates. Asterisks indicate significant pairwise differences using Student’s t-test (*P* ≤ 0.05). (JPG 120 kb)

## Data Availability

The data supporting the results of this article are provided as additional files. All the data pertaining to the present study has been included in the tables/figures of the manuscript. The authors are pleased to share the data upon request.

## Abbreviations

ALDH: Alcohol dehydrogenase
CRY2: CRYPTPCHROME 2
CUAO: Copper amine oxidase
DAF: Days After Flowering
DEG: Differential gene expression
Dw: Dry weight
EVOO: Extra virgin olive oils
FA: Fatty acids
GM: Green mature
GO: Gene ontology
HPLC: High Performance Liquid Chromatography
Ipath: Interactive Pathways
KEGG: Kyoto Encyclopedia of Genes and Genomes
LAMT: S-adenosylmethionine-dependent methyltransferase
Masl: Meters above sea level
MEP: Methylerythritol 4-phosphate
MEP: Plastidic methylerythritol 4-phosphate
MVA: Mevalonate
MVA: Mevalonate
NDHI: NADH dehydrogenase I
*OeFADs*: *FAD* desaturation genes
PAL: Phenylalanine ammonia-lyase
p-HPEA-EA: tyrosol
p-HPEA-EDA: Dialdeid ligustroside aglycon
Phs: Phenolics
PV: Primary oxidation value
qRT-PCR: Quantitative reverse transcriptase polymerase chain reaction
R: Chlorophylls disappearance
RI: Ripening Index
RPKM: Reads Per Kilobase Million
SLS: *Secologanin synthase-like*
TP: Turning purple
VOOs: Virgin olive oils
ß-GLU: Beta-1,3-glucosidase
3-4-DHPEA: Hydroxytirosol
3-4-DHPEA-EDA: Dialdeid oleuropein aglycon
3,4-DHPEA-EA: Elenolic acid linked to hydroxytyrosol
