# Supplementary figures and images for: Changes in gene expression and metabolic profile of drupes of Olea europaea L. cv Carolea in relation to maturation stage and cultivation area
Source: BMC Plant Biol. 2019 Oct 16;19:428. doi: 10.1186/s12870-019-1969-6 (PMC6796363; doi:10.1186/s12870-019-1969-6)

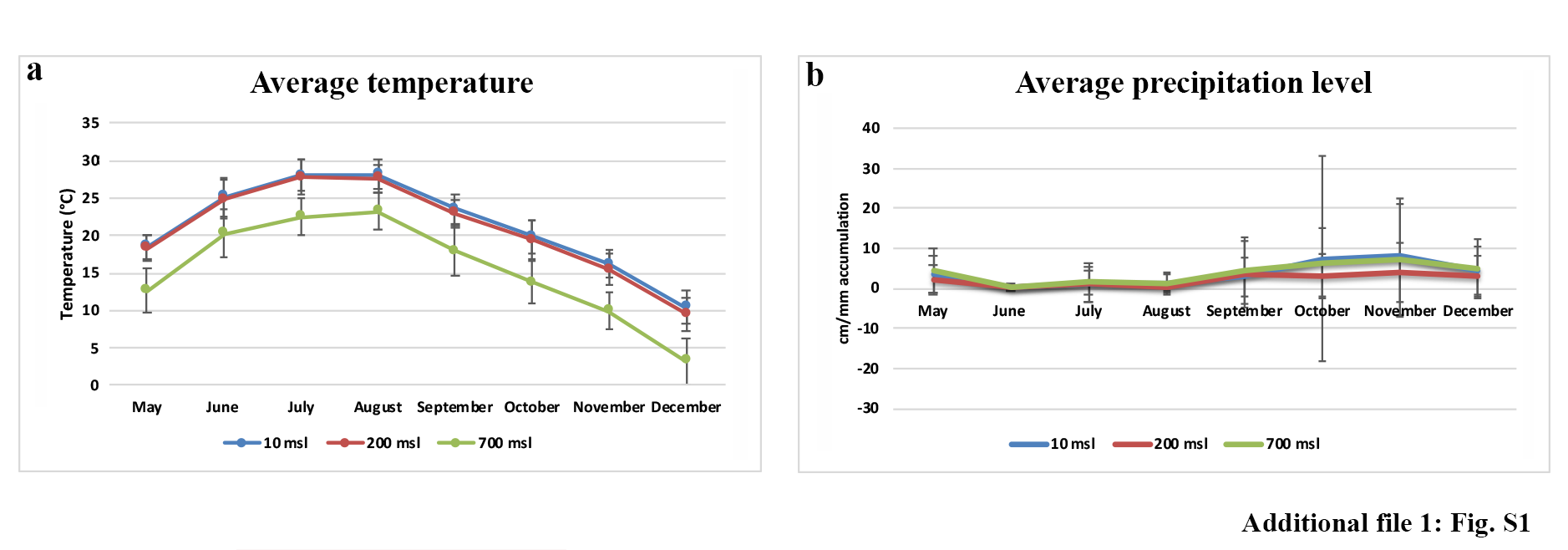

Supplement: Supplementary file 1 — Figure S1. a average temperature and b average precipitation level in the period from May to November 2012 recorded at 10, 200 and 700 masl sites. These informations were obtained by consulting historical databases available on the web (http://www.meteoam.it). (TIF 3637 kb) [file 12870_2019_1969_MOESM1_ESM.tif]

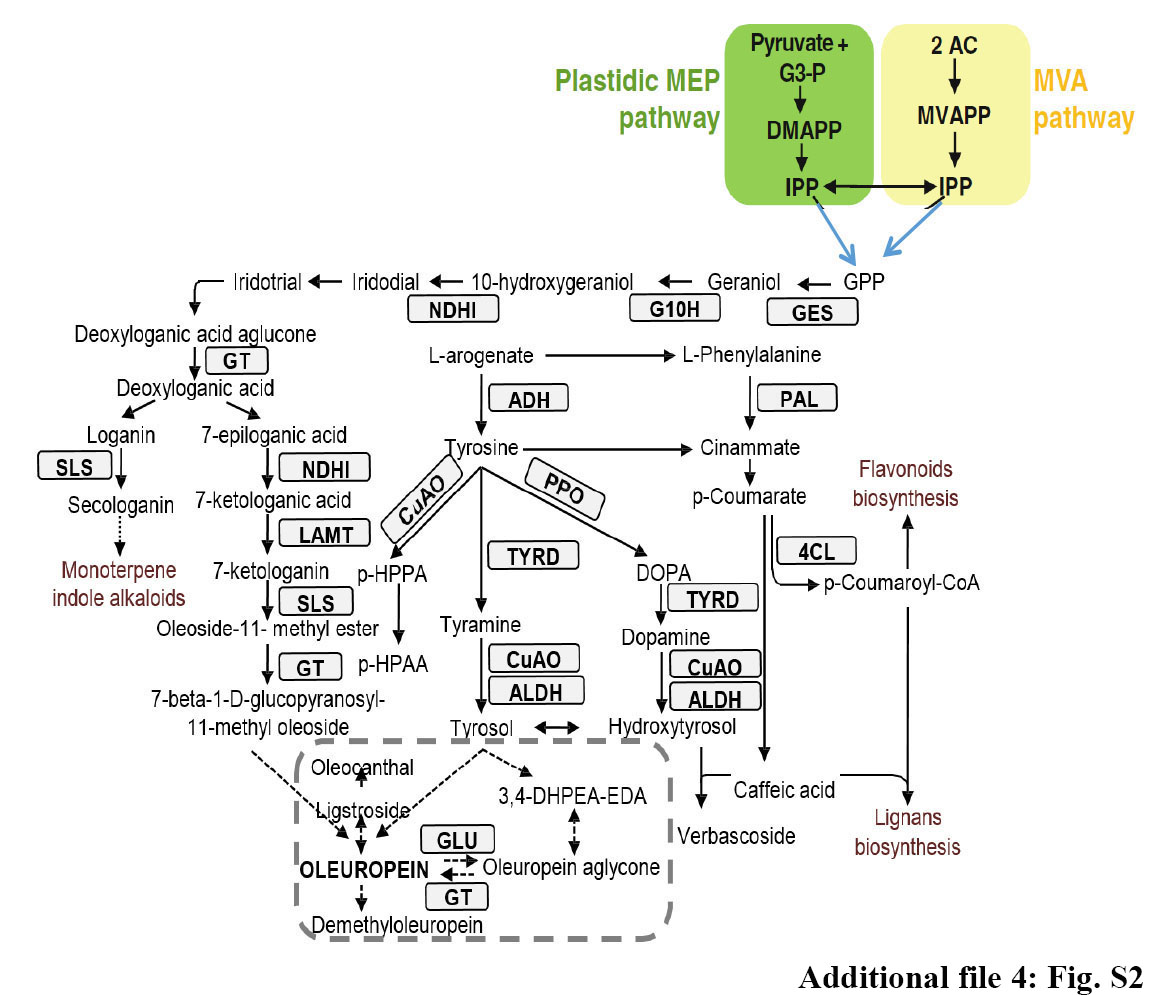

Supplement: Supplementary file 4 — Figure S2. Scheme illustrating the putative biosynthetic pathways of main phenols of olive fruit. (JPG 164 kb) [file 12870_2019_1969_MOESM4_ESM.jpg]

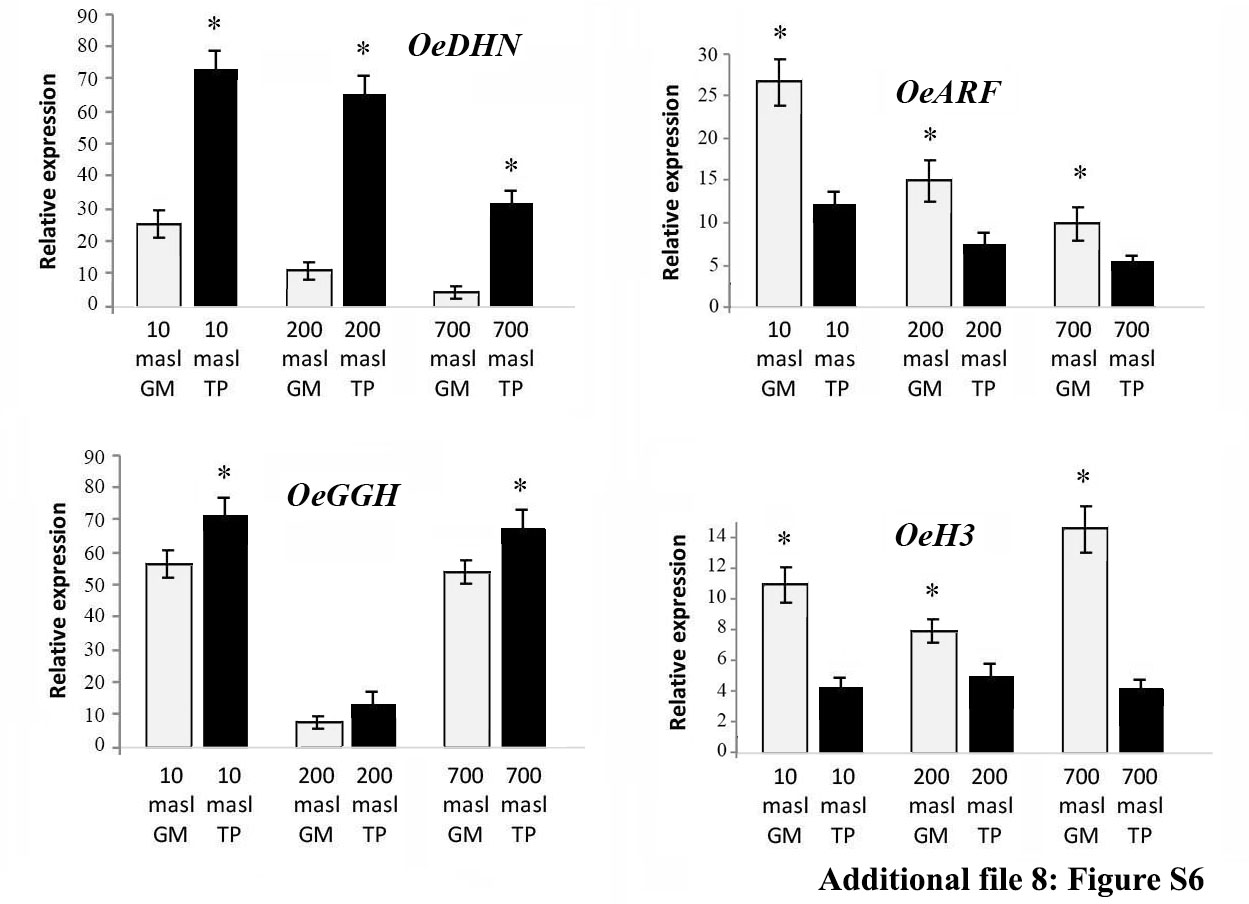

Supplement: Supplementary file 8 — Figure S6. Relative expression levels of OeDHN, OeARF, OeGGH and OeH3 genes in green mature (GM) and turning purple (TP) drupes of ‘Carolea’ populations growing at different meters above sea level (masl), estimated by qRT-PCR after normalization with the ELONGATOR FACTOR 1 alpha (EF1) housekeeping gene. The results were reported as mean values (± standard deviation) of three replicates. Asterisks indicate significant pairwise differences using Student’s t-test (P ≤ 0.05). (JPG 120 kb) [file 12870_2019_1969_MOESM8_ESM.jpg]
