# Supplementary material for: Changes in gene expression and metabolic profile of drupes of Olea europaea L. cv Carolea in relation to maturation stage and cultivation area
Source: BMC Plant Biol. 2019 Oct 16;19:428. doi: 10.1186/s12870-019-1969-6 (PMC6796363; doi:10.1186/s12870-019-1969-6)
Supplement: Supplementary file 2 — Table S1. List of the primers utilized for qRT-PCR analysis. (PPTX 41 kb) [file 12870_2019_1969_MOESM2_ESM.pptx]

## Slide 1
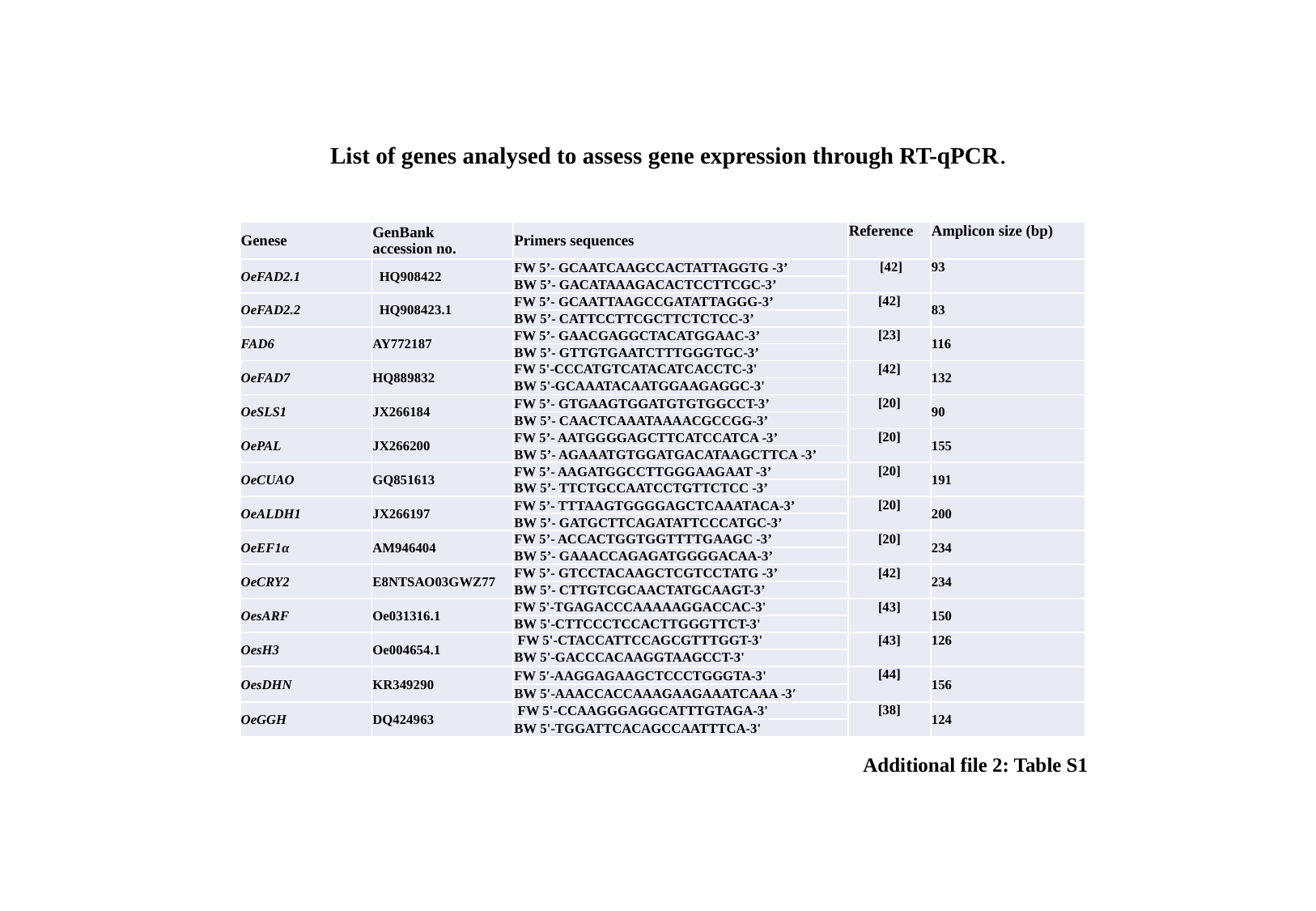

List of genes analysed to assess gene expression through RT-qPCR.
| Genese | GenBank accession no. | Primers sequences | Reference | Amplicon size (bp) |
| --- | --- | --- | --- | --- |
| OeFAD2.1 | HQ908422 | FW 5’- GCAATCAAGCCACTATTAGGTG -3’ | [42] | 93 |
| | | BW 5’- GACATAAAGACACTCCTTCGC-3’ | | |
| OeFAD2.2 | HQ908423.1 | FW 5’- GCAATTAAGCCGATATTAGGG-3’ | [42] | 83 |
| | | BW 5’- CATTCCTTCGCTTCTCTCC-3’ | | |
| FAD6 | AY772187 | FW 5’- GAACGAGGCTACATGGAAC-3’ | [23] | 116 |
| | | BW 5’- GTTGTGAATCTTTGGGTGC-3’ | | |
| OeFAD7 | HQ889832 | FW 5'-CCCATGTCATACATCACCTC-3' | [42] | 132 |
| | | BW 5'-GCAAATACAATGGAAGAGGC-3' | | |
| OeSLS1 | JX266184 | FW 5’- GTGAAGTGGATGTGTGGCCT-3’ | [20] | 90 |
| | | BW 5’- CAACTCAAATAAAACGCCGG-3’ | | |
| OePAL | JX266200 | FW 5’- AATGGGGAGCTTCATCCATCA -3’ | [20] | 155 |
| | | BW 5’- AGAAATGTGGATGACATAAGCTTCA -3’ | | |
| OeCUAO | GQ851613 | FW 5’- AAGATGGCCTTGGGAAGAAT -3’ | [20] | 191 |
| | | BW 5’- TTCTGCCAATCCTGTTCTCC -3’ | | |
| OeALDH1 | JX266197 | FW 5’- TTTAAGTGGGGAGCTCAAATACA-3’ | [20] | 200 |
| | | BW 5’- GATGCTTCAGATATTCCCATGC-3’ | | |
| OeEF1α | AM946404 | FW 5’- ACCACTGGTGGTTTTGAAGC -3’ | [20] | 234 |
| | | BW 5’- GAAACCAGAGATGGGGACAA-3’ | | |
| OeCRY2 | E8NTSAO03GWZ77 | FW 5’- GTCCTACAAGCTCGTCCTATG -3’ | [42] | 234 |
| | | BW 5’- CTTGTCGCAACTATGCAAGT-3’ | | |
| OesARF | Oe031316.1 | FW 5'-TGAGACCCAAAAAGGACCAC-3' | [43] | 150 |
| | | BW 5'-CTTCCCTCCACTTGGGTTCT-3' | | |
| OesH3 | Oe004654.1 | FW 5'-CTACCATTCCAGCGTTTGGT-3' | [43] | 126 |
| | | BW 5'-GACCCACAAGGTAAGCCT-3' | | |
| OesDHN | KR349290 | FW 5′-AAGGAGAAGCTCCCTGGGTA-3' | [44] | 156 |
| | | BW 5′-AAACCACCAAAGAAGAAATCAAA -3′ | | |
| OeGGH | DQ424963 | FW 5'-CCAAGGGAGGCATTTGTAGA-3' | [38] | 124 |
| | | BW 5'-TGGATTCACAGCCAATTTCA-3' | | |
Additional file 2: Table S1
