# Supplementary material for: Changes in gene expression and metabolic profile of drupes of Olea europaea L. cv Carolea in relation to maturation stage and cultivation area
Source: BMC Plant Biol. 2019 Oct 16;19:428. doi: 10.1186/s12870-019-1969-6 (PMC6796363; doi:10.1186/s12870-019-1969-6)
Supplement: Supplementary file 3 — Table S2. Analytical parameters of oil derived from green mature (GM) and turning purple (TP) drupes of ‘Carolea’ populations growing at different meters above sea levels (masl). Significant differences are shown by different letters (P ≤ 0.05) (Students t-test). (PPTX 45 kb) [file 12870_2019_1969_MOESM3_ESM.pptx]

## Slide 1
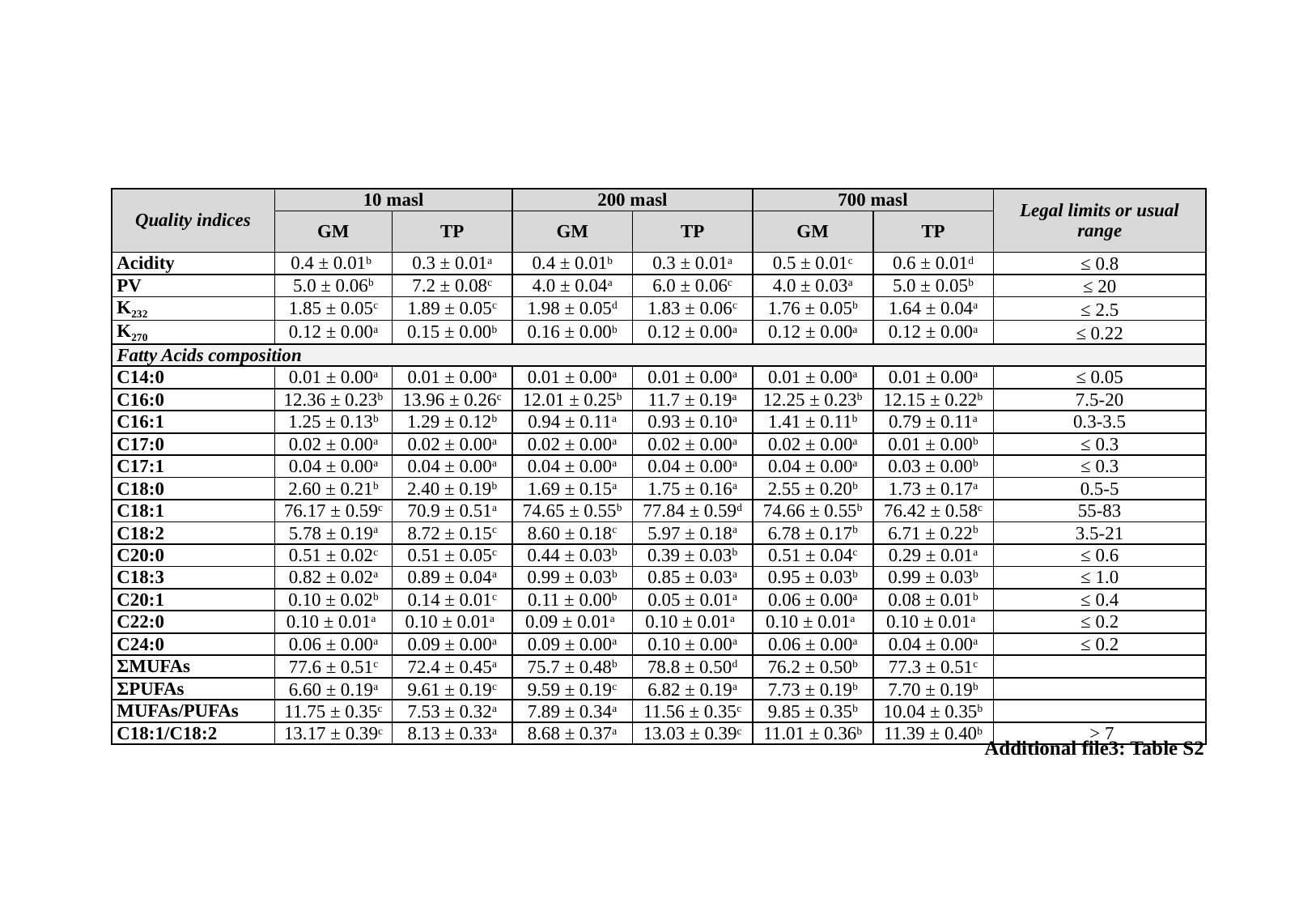

| Quality indices | 10 masl | | 200 masl | | 700 masl | | Legal limits or usual range |
| --- | --- | --- | --- | --- | --- | --- | --- |
| | GM | TP | GM | TP | GM | TP | |
| Acidity | 0.4 ± 0.01b | 0.3 ± 0.01a | 0.4 ± 0.01b | 0.3 ± 0.01a | 0.5 ± 0.01c | 0.6 ± 0.01d | ≤ 0.8 |
| PV | 5.0 ± 0.06b | 7.2 ± 0.08c | 4.0 ± 0.04a | 6.0 ± 0.06c | 4.0 ± 0.03a | 5.0 ± 0.05b | ≤ 20 |
| K232 | 1.85 ± 0.05c | 1.89 ± 0.05c | 1.98 ± 0.05d | 1.83 ± 0.06c | 1.76 ± 0.05b | 1.64 ± 0.04a | ≤ 2.5 |
| K270 | 0.12 ± 0.00a | 0.15 ± 0.00b | 0.16 ± 0.00b | 0.12 ± 0.00a | 0.12 ± 0.00a | 0.12 ± 0.00a | ≤ 0.22 |
| Fatty Acids composition | | | | | | | |
| C14:0 | 0.01 ± 0.00a | 0.01 ± 0.00a | 0.01 ± 0.00a | 0.01 ± 0.00a | 0.01 ± 0.00a | 0.01 ± 0.00a | ≤ 0.05 |
| C16:0 | 12.36 ± 0.23b | 13.96 ± 0.26c | 12.01 ± 0.25b | 11.7 ± 0.19a | 12.25 ± 0.23b | 12.15 ± 0.22b | 7.5-20 |
| C16:1 | 1.25 ± 0.13b | 1.29 ± 0.12b | 0.94 ± 0.11a | 0.93 ± 0.10a | 1.41 ± 0.11b | 0.79 ± 0.11a | 0.3-3.5 |
| C17:0 | 0.02 ± 0.00a | 0.02 ± 0.00a | 0.02 ± 0.00a | 0.02 ± 0.00a | 0.02 ± 0.00a | 0.01 ± 0.00b | ≤ 0.3 |
| C17:1 | 0.04 ± 0.00a | 0.04 ± 0.00a | 0.04 ± 0.00a | 0.04 ± 0.00a | 0.04 ± 0.00a | 0.03 ± 0.00b | ≤ 0.3 |
| C18:0 | 2.60 ± 0.21b | 2.40 ± 0.19b | 1.69 ± 0.15a | 1.75 ± 0.16a | 2.55 ± 0.20b | 1.73 ± 0.17a | 0.5-5 |
| C18:1 | 76.17 ± 0.59c | 70.9 ± 0.51a | 74.65 ± 0.55b | 77.84 ± 0.59d | 74.66 ± 0.55b | 76.42 ± 0.58c | 55-83 |
| C18:2 | 5.78 ± 0.19a | 8.72 ± 0.15c | 8.60 ± 0.18c | 5.97 ± 0.18a | 6.78 ± 0.17b | 6.71 ± 0.22b | 3.5-21 |
| C20:0 | 0.51 ± 0.02c | 0.51 ± 0.05c | 0.44 ± 0.03b | 0.39 ± 0.03b | 0.51 ± 0.04c | 0.29 ± 0.01a | ≤ 0.6 |
| C18:3 | 0.82 ± 0.02a | 0.89 ± 0.04a | 0.99 ± 0.03b | 0.85 ± 0.03a | 0.95 ± 0.03b | 0.99 ± 0.03b | ≤ 1.0 |
| C20:1 | 0.10 ± 0.02b | 0.14 ± 0.01c | 0.11 ± 0.00b | 0.05 ± 0.01a | 0.06 ± 0.00a | 0.08 ± 0.01b | ≤ 0.4 |
| C22:0 | 0.10 ± 0.01a | 0.10 ± 0.01a | 0.09 ± 0.01a | 0.10 ± 0.01a | 0.10 ± 0.01a | 0.10 ± 0.01a | ≤ 0.2 |
| C24:0 | 0.06 ± 0.00a | 0.09 ± 0.00a | 0.09 ± 0.00a | 0.10 ± 0.00a | 0.06 ± 0.00a | 0.04 ± 0.00a | ≤ 0.2 |
| ΣMUFAs | 77.6 ± 0.51c | 72.4 ± 0.45a | 75.7 ± 0.48b | 78.8 ± 0.50d | 76.2 ± 0.50b | 77.3 ± 0.51c | |
| ΣPUFAs | 6.60 ± 0.19a | 9.61 ± 0.19c | 9.59 ± 0.19c | 6.82 ± 0.19a | 7.73 ± 0.19b | 7.70 ± 0.19b | |
| MUFAs/PUFAs | 11.75 ± 0.35c | 7.53 ± 0.32a | 7.89 ± 0.34a | 11.56 ± 0.35c | 9.85 ± 0.35b | 10.04 ± 0.35b | |
| C18:1/C18:2 | 13.17 ± 0.39c | 8.13 ± 0.33a | 8.68 ± 0.37a | 13.03 ± 0.39c | 11.01 ± 0.36b | 11.39 ± 0.40b | > 7 |
Additional file3: Table S2
