# Supplementary material for: Changes in gene expression and metabolic profile of drupes of Olea europaea L. cv Carolea in relation to maturation stage and cultivation area
Source: BMC Plant Biol. 2019 Oct 16;19:428. doi: 10.1186/s12870-019-1969-6 (PMC6796363; doi:10.1186/s12870-019-1969-6)
Supplement: Supplementary file 5 — Figure S3. Interactive pathways analysis during drupe maturation of ‘Carolea’ population growing at 10 masl. The red and blue lines indicate the up and down regulated pathways respectively. (PPTX 920 kb) [file 12870_2019_1969_MOESM5_ESM.pptx]

## Slide 1
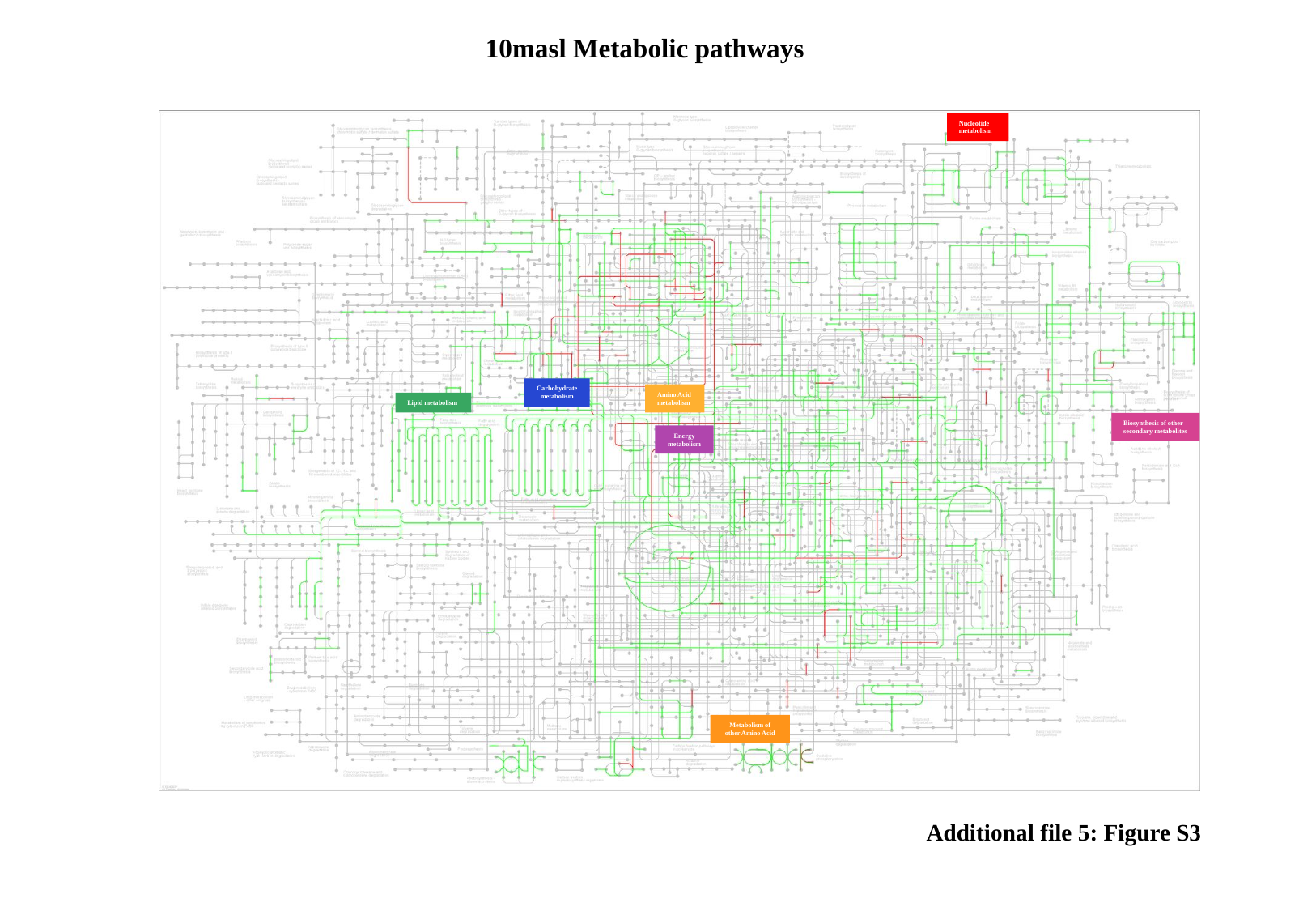

10masl Metabolic pathways
Nucleotide metabolism
Carbohydrate metabolism
Amino Acid metabolism
Lipid metabolism
Biosynthesis of other secondary metabolites
Energy metabolism
Metabolism of other Amino Acid
Additional file 5: Figure S3
