# Supplementary material for: Changes in gene expression and metabolic profile of drupes of Olea europaea L. cv Carolea in relation to maturation stage and cultivation area
Source: BMC Plant Biol. 2019 Oct 16;19:428. doi: 10.1186/s12870-019-1969-6 (PMC6796363; doi:10.1186/s12870-019-1969-6)
Supplement: Supplementary file 6 — Figure S4. Interactive pathways analysis during drupe maturation of ‘Carolea’ population growing at 200 masl. The red and blue lines indicate the up and down regulated pathways respectively. (PPTX 935 kb) [file 12870_2019_1969_MOESM6_ESM.pptx]

## Slide 1
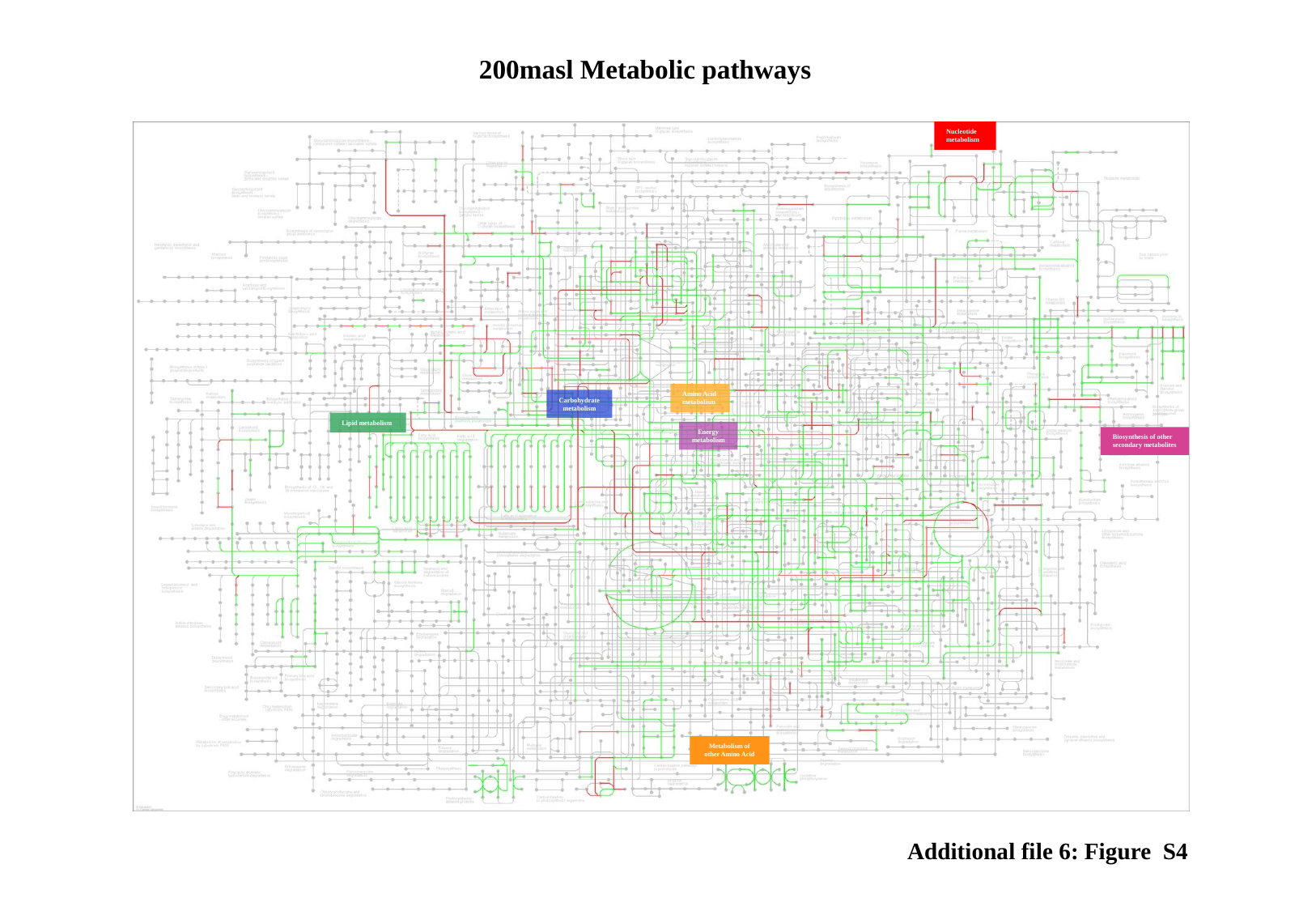

200masl Metabolic pathways
Nucleotide metabolism
Amino Acid metabolism
Carbohydrate metabolism
Lipid metabolism
Energy metabolism
Biosynthesis of other secondary metabolites
Metabolism of other Amino Acid
Additional file 6: Figure S4
